# Association of Unemployment and Government Stringency With the Increased Burden of Anxiety and Depression Amid the Public Health Emergency: A Global Perspective

**DOI:** 10.1002/brb3.71132

**Published:** 2025-12-10

**Authors:** Chen Zhao, Xīn Gào, Mengdi Zhang, Weihua Yue, Xiao Zhang

**Affiliations:** ^1^ Department of Neurology Peking University Third Hospital Beijing China; ^2^ Beijing Key Laboratory of Biomarker and Translational Research in Neurodegenerative Diseases Beijing China; ^3^ Key Laboratory for Neuroscience National Health Commission/Ministry of Education, Peking University Beijing China; ^4^ Department of Epidemiology and Biostatistics, School of Public Health Peking University Beijing China; ^5^ Key Laboratory of Epidemiology of Major Diseases Peking University, Ministry of Education Beijing China; ^6^ Institute of Mental Health, National Clinical Research Center For Mental Disorders Peking University Sixth Hospital Beijing China; ^7^ NHC Key Laboratory of Mental Health (Peking University) Beijing China; ^8^ PKU‐IDG/McGovern Institute for Brain Research of Peking University Beijing China; ^9^ Research Unit of Diagnosis and Treatment of Mood Cognitive Disorder (2018RU006) Chinese Academy of Medical Sciences Beijing China; ^10^ Chinese Institute for Brain Research Beijing China

**Keywords:** anxiety disorders, COVID‐19, depressive disorders, government stringency index, unemployment

## Abstract

**Background:**

The COVID‐19 pandemic has coincided with marked increases in anxiety and depressive disorders. Beyond infection risks, socioeconomic stressors such as rising unemployment and stringent government responses may have contributed substantially to these burdens but remain poorly quantified globally.

**Methods:**

We conducted an ecological country‐level cross‐sectional study using data from the Global Burden of Disease 2021 database, World Bank Open Data, and Our World in Data, covering 169 countries and territories. Primary outcomes were changes in age‐standardized incidence rates (ASIRs) of anxiety and depressive disorders between 2019 and 2020. Exposures included changes in unemployment rates and the government stringency index (GSI) during the same period. Associations were examined overall and stratified by sex, age, and sociodemographic index (SDI).

**Results:**

Globally, ASIRs of anxiety and depressive disorders increased in 2020 compared with 2019. Median increases were larger in high‐ than low‐SDI countries for anxiety (17.12% vs. 11.2%) and depressive disorders (17.04% vs. 9.04%). Greater increases in unemployment were associated with larger increases in ASIR of anxiety (*β* = 1.43, 95% CI: 0.56–2.29) and depressive disorders (*β* = 1.70, 95% CI: 0.78–2.62). Higher GSI was also positively associated with increases in ASIR of anxiety (*β* = 1.08, 95% CI: 0.19–1.98) and depressive disorders (*β* = 1.34, 95% CI: 0.41–2.28). Associations were stronger in females than males and most pronounced in children and adolescents.

**Conclusion:**

Rising unemployment and stringent government responses were significantly associated with the global increase in anxiety and depressive disorders during the COVID‐19 pandemic, highlighting the need for future public health strategies that balance infection control with economic protections and mental health support

## Graphical abstract

This ecological, country‐level cross‐sectional study found that greater increases in unemployment and higher government stringency were significantly associated with rises in the incidence of anxiety and depressive disorders, particularly among females, younger populations, and countries with higher sociodemographic indices.

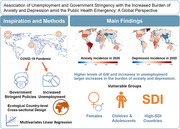

## 1 Introduction

Anxiety and depressive disorders are among the most prevalent mental health conditions globally. Over the past three decades, they have imposed a growing incidence (GBD 2021 Diseases and Injuries Collaborators 2024). During the COVID‐19 pandemic, this upward trajectory was significantly amplified, with anxiety and depressive disorders emerging as primary drivers of the escalating global disease burden (C. Chen, Zhou, et al. 2025; Santomauro et al. 2021). In 2021, anxiety and depressive disorders accounted for approximately 54 million and 357 million new cases worldwide, ranking as the second and sixth leading contributors to global disability‐adjusted life years (DALYs), respectively (GBD 2021 Diseases and Injuries Collaborators 2024; S. Chen, Huang, et al. 2025; J. Zhou et al. 2025). The sustained rise in cases has exerted growing pressure not only on health care systems but also on broader societal and economic functioning. Beyond their direct mental health impacts, anxiety and depressive disorders confer far‐reaching consequences, including but not limited to elevated risks of comorbid physical illnesses and premature mortality (Sariaslan et al. 2022), reduced educational attainment (Dalsgaard et al. 2020), constrained occupational opportunities (Bruggeman et al. 2024), and social isolation (Holt‐Lunstad 2024).

Mental health and these socioeconomic factors are interlinked in a bidirectional manner (Kirkbride et al. 2024). A substantial body of epidemiological evidence has shown that socioeconomic disadvantage strongly influences mental illness risk over the life course, involving multiple interconnected dimensions such as family income, education, occupation, and living conditions (Kirkbride et al. 2024). Unemployment, for instance, often precipitates financial hardship, leading to chronic stress, uncertainty, poor living conditions, and increased risks of illness, trauma, and violence (Thomson et al. 2022). Beyond financial strain, unemployment can disrupt daily routines and erode one's sense of purpose, thereby compounding the risk of developing anxiety and depressive disorders (Ridley et al. 2020). These socioeconomic risks are further exacerbated during public health emergencies, which may amplify mental health disparities (Y. Zhou et al. 2021). It has been shown that the COVID‐19 pandemic triggered an unprecedented global economic downturn and historically high unemployment rates across developed and developing nations (Tang and Abosedra 2023; Fan et al. 2025). Moreover, government policies implemented in response to COVID‐19, such as lockdowns and social distancing measures, can disrupt social networks, limit access to mental health services, and exacerbate feelings of isolation. This dramatic increase in the burden of anxiety and depressive disorders suggests that these diseases are particularly susceptible to pandemic‐related stressors (Moreno et al. 2020). While a growing number of studies have reported significant associations between the socioeconomic factors and poor mental health outcomes during the COVID‐19 pandemic (Xiong et al. 2020; Penninx et al. 2022), most have been restricted to single‐country analyses and predominantly rely on self‐reported symptoms, limiting their cross‐national comparability and generalizability. It remains unclear how these extensive policy changes affect individuals of different ages and genders across nations with varying policies, which is a topic worthy of further exploration.

Identifying the determinants of rising anxiety and depression burden is essential for targeted prevention and strengthening future crisis preparedness. There is a clear need for a comprehensive global assessment that links the disease burden with internationally comparable measures of economic disruption and government response. In this study, we integrated Global Burden of Disease (GBD) estimates for anxiety and depressive disorders with country‐level socioeconomic indicators from the Oxford COVID‐19 Government Response Tracker and World Bank Open Data. We examined whether changes in unemployment and the intensity of government restrictions are associated with increases in age‐standardized incidence or prevalence of anxiety and depressive disorders during the initial pandemic period using an ecological cross‐country design.

## 2 Materials and Methods

### 2.1 Data Sources

We conducted an ecological, country‐level cross‐sectional analysis to examine the associations between changes in socioeconomic indicators and mental health burden during the COVID‐19 pandemic using various databases. The GBD is a comprehensive epidemiological database coordinated by the Institute for Health Metrics and Evaluation (IHME) (GBD Collaborative Network 2024). In GBD 2021, the burden of 371 diseases and injuries was estimated for 204 countries and territories using data from 100,983 sources (GBD 2021 Diseases and Injuries Collaborators 2024). We obtained country‐level data on the measures of disease burden of anxiety disorders, depressive disorders, and COVID‐19, sociodemographic index (SDI), and health workforce density (per 10,000 population) from the GBD 2021 database. Our World in Data (OWID) is an online scientific publication covering topics on poverty, public health, education, climate change, and other critical issues (Herre et al. 2024). This study extracted data on the government stringency index (GSI) in both 2020 and 2021 from OWID (Hale et al. 2021). Unemployment rates and health expenditure per capita were sourced from the World Bank Open Data.

### 2.2 Disease Definitions

Anxiety disorders were modeled as a single cause for any subtypes of anxiety disorder defined according to diagnostic criteria presented in the Diagnostic and Statistical Manual of Mental Disorders (DSM‐IV‐TR: 300.0‐300.3, 208.3, 309.21, 309.81) and the 10th International Classification of Diseases and Related Health Problems (ICD‐10: F40‐42, F43.0, F43.1, F93.0‐93.2, F93.8). Depressive disorders are categorized to include major depressive disorder (DSM‐IV‐TR: 296.21‐24, 296.31‐34; ICD‐10: F32.0‐9, F33.0‐9) and dysthymia (DSM‐IV‐TR: 300.4; ICD‐10: F34.1).

### 2.3 Disease Burden Measures

Percentage changes of age‐standardized incidence rate (ASIR) of anxiety disorders and depressive disorders between 2019 and 2020 were calculated as the primary outcomes.

Incidence of anxiety and depressive disorders was modeled using the Disease Modelling Meta‐Regression tool (version 2.1), which is a Bayesian disease modeling meta‐regression tool synthesizing various available data (GBD 2021 Diseases and Injuries Collaborators 2024). The ASIRs for specific age groups (< 20, 20–≤ 64, and > 65) were recalculated using the following steps: First, crude incidence rates of each 5‐year age stratum were obtained from the GBD database. Second, the crude incidence rate was multiplied by the proportion of the standard reference population in the corresponding age stratum. These products were then summed across all 5‐year age groups within the specific age group. Additionally, years lived with disability (YLD) were used to quantify the burden of mental health conditions that result in nonfatal health outcomes. For anxiety and depressive disorders, YLD is equivalent to DALYs in the GBD study.

### 2.4 Socioeconomic Indicators

Unemployment rate is defined as the percentage of the labor force actively seeking work but without employment, based on the International Labour Organization's modeled estimates and projections database. Changes in this rate from 2019 to 2020 were used as the exposure. The median level of GSI for 2020 was used to quantify the strictness of COVID‐19 containment policies across countries. SDI was utilized as an integrated indicator of a location's socioeconomic development. Additionally, health workforce density (per 10,000 population) and health expenditure per capita were included to reflect the availability of health care resources during the pandemic.

### 2.5 Statistical Analysis

#### 2.5.1 Descriptive Statistics

Descriptive analyses were conducted to characterize the global nonfatal burden of anxiety and depressive disorders. The age and gender distributions for the year 2021 were depicted through population pyramids, and heatmaps were utilized to represent the evolution of age‐specific burden over time. Socioeconomic indicators were presented as median and interquartile range (IQR). Data preprocessing, descriptive statistics, and data visualization were performed using R software (version 4.2.3).

#### 2.5.2 Inequality by SDI Levels

The slope index of inequality and the concentration index were used to quantify mental disorder burden inequality across 204 countries and territories, with the SDI representing socioeconomic status. The slope index of inequality is derived from the slope of a linear regression model that relates country‐level age‐standardized incidence and YLDs to the median of SDI quantiles. A positive (or negative) slope indicates that disease burden rises (or falls) with higher SDI. The concentration index (ranging from −1 to 1) measures inequality by calculating twice the area between the line of equality and the Lorenz curve, which plots cumulative incidence and YLDs ranked by SDI. A value closer to 1 or −1 indicates a concentration of burden in higher or lower SDI countries, respectively. Values closer to 0 indicate a more equitable (or even) distribution across all SDI levels.

#### 2.5.3 Generalized Linear Model

A matrix of Spearman's correlation coefficients (rs) was generated to visualize the pairwise correlations among percentage changes of anxiety and depressive disorders and socioeconomic indicators. Generalized linear models (GLIMs) with Gaussian distributions were used to assess associations. Model diagnostics and robustness checks were performed to ensure the validity of GLIMs. Residual normality of dependent variables was assessed using histograms and *Q*–*Q* plots (Figures S1 and S2). Following rigorous data quality assessment criteria, 169 of the initial 204 countries and territories were retained for final analysis. The adjustments of GLIM models were determined using directed acyclic graphs (Figures S3 and S4). Additionally, variance inflation factors (VIFs) were calculated to evaluate multicollinearity among covariates. VIFs of covariates were < 5, indicating acceptable levels of correlation. All continuous variables (exposures and covariates) were standardized to *z*‐scores (mean = 0, standard deviation [SD] = 1) to facilitate comparison of effect sizes. The model of unemployment rate changes was adjusted for COVID‐19 ASIR, SDI, median age, female population proportion, and GSI. The model of GSI was adjusted for COVID‐19 ASIR, SDI, health expenditure per capita, and health workforce density. All independent variables were standardized to *z*‐scores to have a mean of 0 and SD of 1. Two‐sided hypothesis tests were conducted with a statistical significance level of 0.05.

## 3 Results

### 3.1 Age and Sex Patterns of Anxiety and Depressive Disorders

**FIGURE 1:**
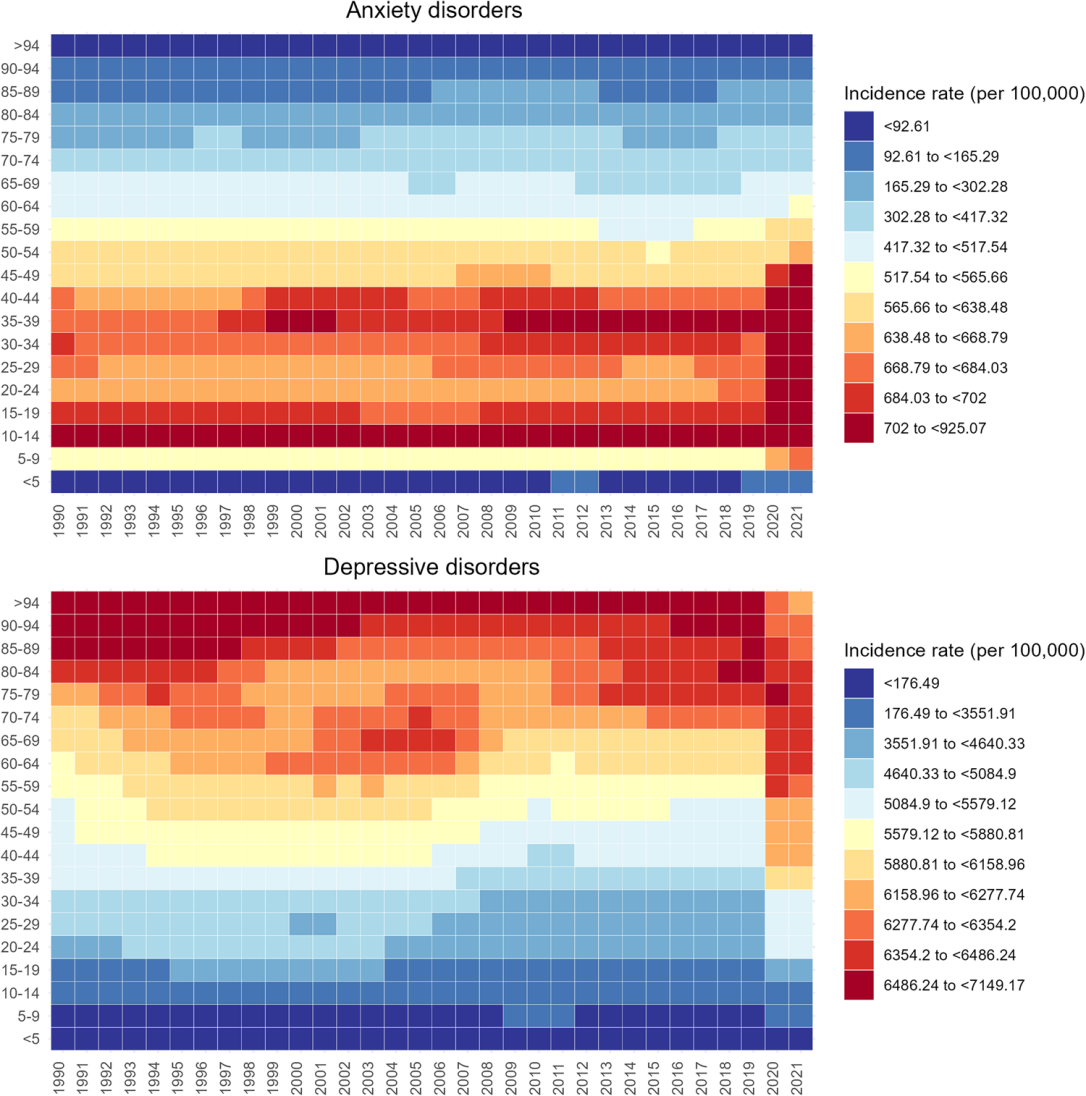
Heatmap of incidence rate of anxiety and depressive disorders by age from 1990 to 2021.

Over the past three decades, the incidence rate of anxiety disorders was particularly high among adolescents and adults ≤ 60 years, while depressive disorders showed a greater burden among the elderly population (Figure 1). Females exhibited higher incidence rates than males in all age groups for anxiety and depressive disorders (Figure S5). During the COVID‐19 pandemic, the increase in the burden of anxiety and depressive disorders exceeded the cumulative changes over the previous three decades. Notably, the incidence of both disorders rose more steeply among younger individuals than older adults (Figure S6).

### 3.2 Inequities of Anxiety and Depressive Disorders Across SDI Levels

**FIGURE 2:**
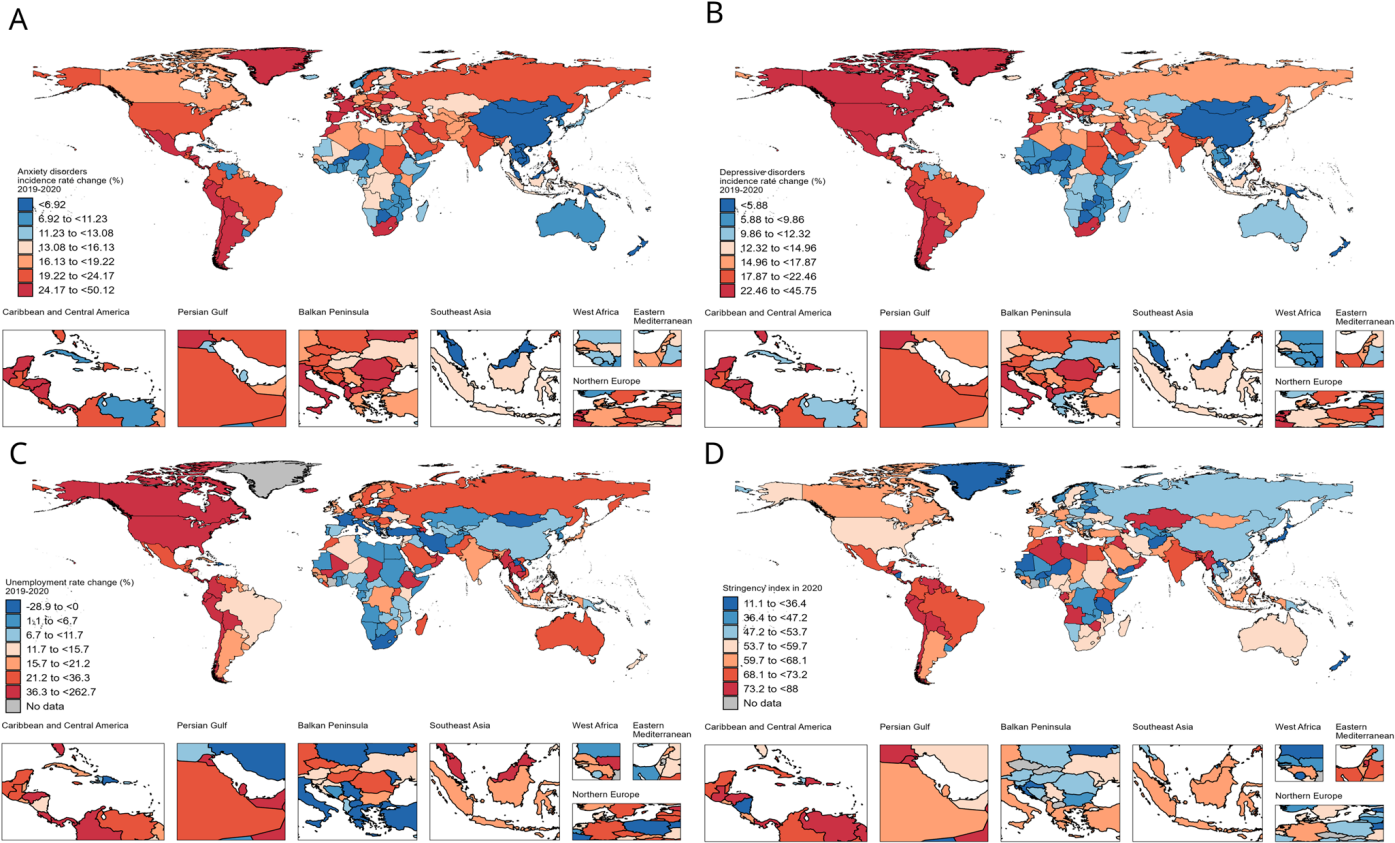
Geographic distributions of the percentage change in incidence rate of (A) anxiety disorders and (B) depressive disorders from 2019 to 2020, (C) percentage change in unemployment rate from 2019 to 2020, and (D) government stringency index in 2020.

Figure 2 presents the health inequities across different levels of the SDI for anxiety and depressive disorders. For anxiety disorders, the incidence rate showed an increase with higher SDI levels. It had a concentration index of 0.07 in 1990, which slightly decreased to 0.06 in 2021. Depressive disorders displayed a negative CI, suggesting a higher burden in more disadvantaged groups. The concentration index remained unchanged between 1990 and 2021.

**FIGURE 3:**
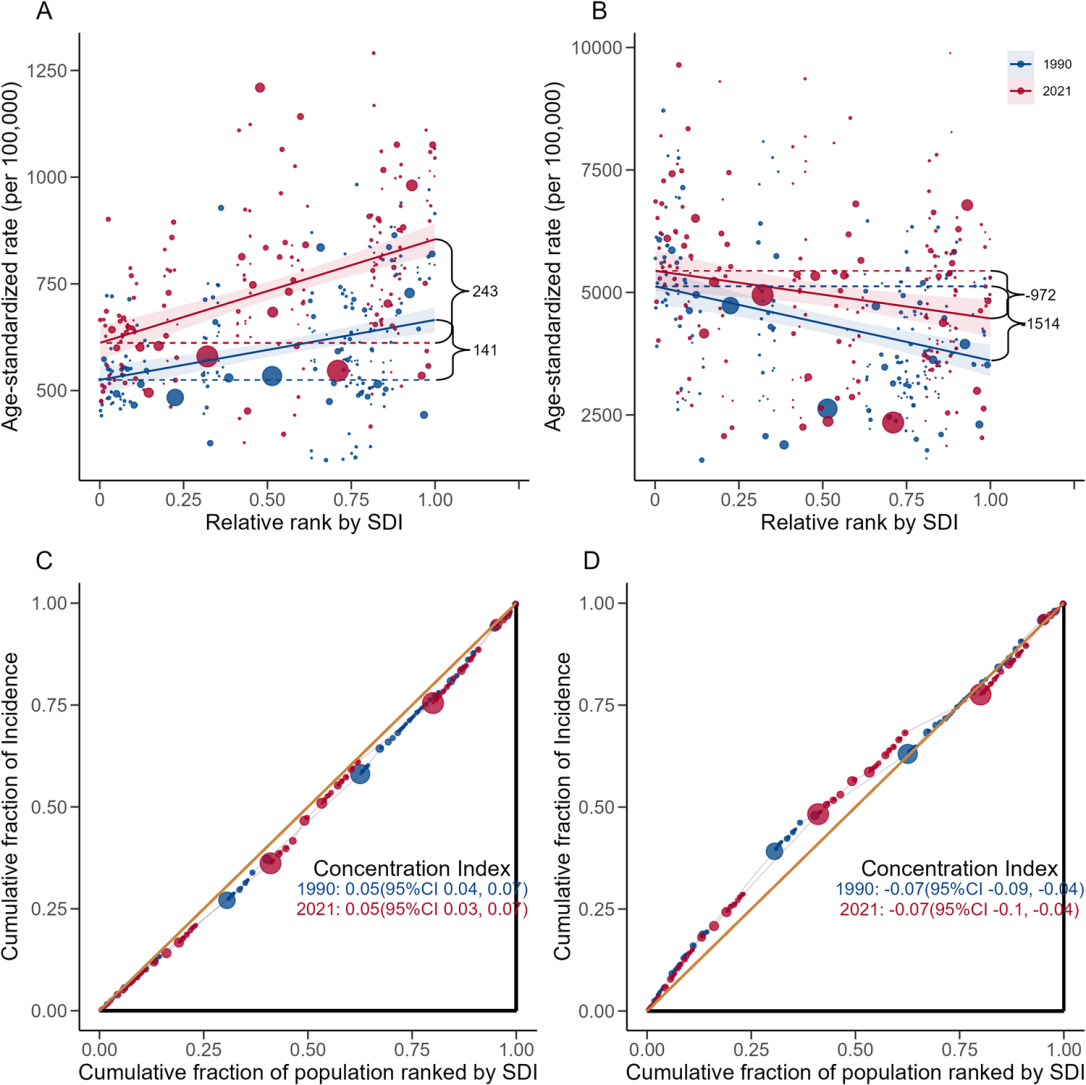
The slope and concentration indices of inequality for incidence rates of anxiety and depressive disorders by social‐demographic index level, 1990 and 2021. (A) Absolute inequality for anxiety disorders, (B) absolute inequality for depressive disorders, (C) relative inequality for anxiety disorders, and (D) relative inequality for depressive disorders.

Between 2019 and 2020, the changes in ASIR of anxiety disorders were positively correlated with SDI levels, rising from a median of 11.2% (IQR: 8.23–13.25) in low‐SDI countries to 17.12% (11.88–21.29) in high‐SDI countries. A similar trend was observed for depressive disorders, with ASIR increasing from 9.04% (7.34–10.34) in low‐SDI countries to 17.04% (12.78–22.59) in high‐SDI countries (Table 1). Geographically, Europe, North America, Latin America, and the Middle East and North Africa exhibited the highest increases in anxiety and depression incidence rates during the first year of the pandemic (Figure 3).

**TABLE 1:** Medians (interquartile ranges) of changes in anxiety disorders and depressive disorders incidence rate, COVID‐19 incidence rate, demographic, and socioeconomic indicator changes by SDI levels in 2020.

	Low SDI		Low‐middle SDI		Middle SDI		High‐middle SDI			High SDI	
	*n*	Median (IQR)	*n*	Median (IQR)	*n*	Median (IQR)	*n*	Median (IQR)		*n*	Median (IQR)
Anxiety disorders incidence rate change (%), 2019–2020	33	11.2 (8.23–13.25)	44	13.23 (8.21–20.33)	40	16.16 (11.71–21.85)	47	16.42 (12.13–21.75)		39	17.12 (11.88–21.29)
Depressive disorder incidence rate change (%), 2019–2020	33	9.04 (7.34–10.34)	44	11.32 (7.35–18.63)	40	15.96 (12.18–18.79)	47	15.46 (11.66–18.99)		39	17.04 (12.78–22.59)
Median age of population (years)	33	17.7 (16.19–18.59)	38	22.62 (20.16–25.28)	36	29.15 (26.49–31.96)	43	37.13 (32.23–40.81)		39	41.39 (37.36–42.98)
Female percentage of population (%)	33	50.22 (49.75–50.59)	38	50.17 (49.68–50.5)	36	50.26 (49.87–50.64)	43	51.17 (49.89–51.98)		38	50.4 (49.64–50.93)
COVID‐19 age‐standardized incidence rate (per 100,000)	33	25,788 (15,631–37,237)	44	24,986 (4684–33,063)	40	17,742 (3670–32,085)	47	12,061 (1877–25,988)		39	8636 (3435–17,472)
Unemployment rate change (%), 2019–2020	33	11.63 (6.75–19.43)	40	10.91 (5.61–21.14)	37	14.75 (6.99–27.73)	38	15.45 (2.06–20.9)		33	21.02 (12.34–32.26)
Median stringency index^a^, 2020	32	43.75 (35.57–54.09)	37	56.55 (42.86–64.88)	32	60.71 (53.57–65.26)	38	56.1 (46.21–65.85)		39	50.6 (43.75–58.49)
Health workforce density (per 10,000), 2019	33	26.98 (21.44–34.41)	44	53.14 (43.72–70.89)	40	109.17 (78.53–154.44)	47	204.8 (143.75–249.46)		39	385.46 (300.73–501.34)
Health expenditure per capita ($), 2020	33	133 (105–198)	44	391 (216–631)	39	1073 (746–1424)	47	1830 (1222.5–2616.5)		38	6375.5 (4197.5–7329.75)

*Note*: Sociodemographic index was the mean values of SDI of each country and territory in 2020.

Abbreviations: IQR, interquartile range; SDI, sociodemographic index.

^a^ Government stringency index were the median values of each county and territory throughout 2020. COVID‐19 testing policies, COVID‐19 contact tracing policies, face covering policies, public events cancellation, public gathering cancellation, public transport restrictions, school closures, and workplace closures were the mode values throughout 2020.

### 3.3 Socioeconomic Indicators During the COVID‐19 Pandemic

Table 1 presents medians and IQRs of key socioeconomic indicators across different SDI levels in 2020. Median age of the whole population increased from 17.7 years (IQR: 16.19–18.59) in low SDI regions to 41.39 years (37.36–42.98) in high SDI regions. The increase in unemployment rate was the highest in high‐SDI regions, with a Spearman correlation coefficient of 0.15 between SDI levels and the increase in unemployment rate (Figure S9). The association between the GSI and the SDI demonstrated an inverted U‐shaped pattern. Specifically, regions with middle levels of SDI exhibited the highest GSI values (60.71 [53.57–65.26]). Health workforce density varied from 26.98 per 10,000 population (21.44–34.41) in low SDI regions to 385.46 per 10,000 population (300.73–501.34) in high SDI regions. Health expenditure per capita also increased with SDI level, from $133 (105–198) in low SDI regions to $6375.5 (4197.5–7329.75) in high SDI regions.

### 3.4 Association Between Socioeconomic Indicators and Mental Disorders

**FIGURE 4:**
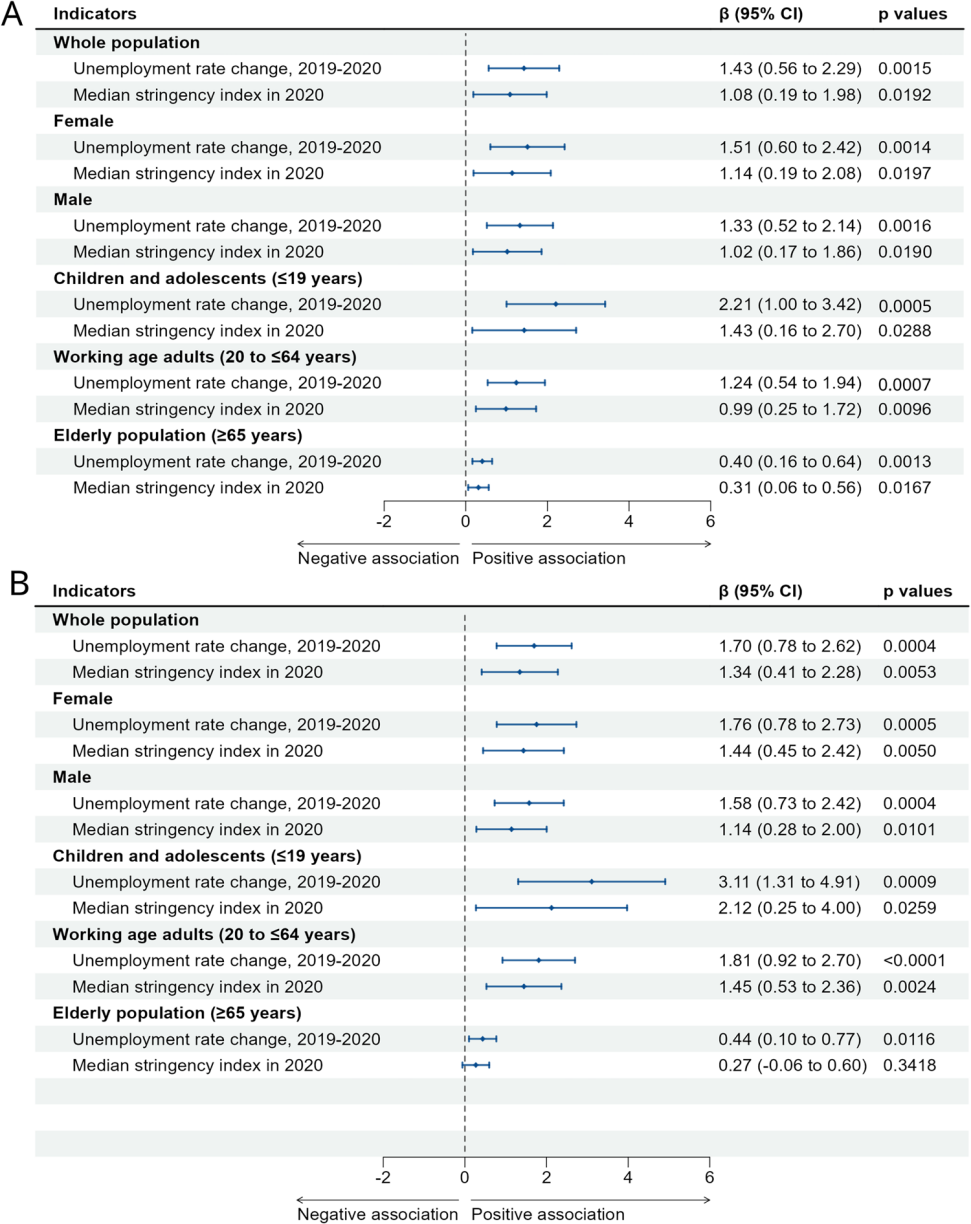
Nation‐level associations of percentage change in incidence rate of mental disorders with unemployment rate changes and stringency during the pandemic, overall, by sex, and by age group. (A) Anxiety disorders and (B) depressive disorders.

Both unemployment and government stringency were significantly associated with higher ASIRs of anxiety and depressive disorders. At the population level, each one‐SD increase in the unemployment rate corresponded to regression coefficients of 1.43 (95% CI: 0.56–2.29) for anxiety and 1.70 (0.78–2.62) for depressive disorders (Figure 4). Similarly, a one‐SD increase in the GSI was associated with coefficients of 1.08 (95% CI: 0.19–1.98) for anxiety and 1.34 (0.41–2.28) for depressive disorders (Figure 4).

Stratified analyses revealed stronger associations in females. For unemployment, coefficients were 1.51 (0.60–2.42) for anxiety and 1.76 (0.78–2.73) for depressive disorders in females, compared with 1.33 (0.52–2.14) and 1.58 (0.73–2.42), respectively, in males. For GSI, the coefficients for depressive disorders were 1.44 (95% CI: 0.45–2.42) in females and 1.14 (95% CI: 0.28–2.00) in males. For anxiety disorders, the corresponding coefficients were 1.04 (95% CI: 0.19–2.08) in females and 1.02 (95% CI: 0.17–1.86) in males.

Age‐stratified analyses indicated the greatest vulnerability among children and adolescents. For unemployment, coefficients reached 2.21 (1.00–3.42) for anxiety and 3.11 (1.31–4.91) for depressive disorders, substantially higher than in working‐age adults (anxiety disorders: 1.24 [0.54–1.94]; depressive disorders: 1.81 [0.92–2.70]) or the elderly (anxiety disorders: 0.40 [0.16–0.64]; depressive disorders: 0.44 [0.10–0.77]). For GSI, children and adolescents also showed the strongest associations (anxiety disorders: 1.43 [0.16–2.70]; depressive disorders: 2.12 [0.27–3.97]), while the elderly exhibited weak or nonsignificant effects, particularly for depressive disorders (0.27, 95% CI: –0.06 to 0.60).

## 4 Discussion

In this cross‐sectional analysis using nation‐level data, we found that the incidence of anxiety disorders was disproportionately higher among younger individuals, whereas depressive disorders exerted a greater burden among the elderly. Across all age groups, females consistently exhibited higher incidence rates than males. Socioeconomic inequalities displayed heterogeneous patterns. The burden of anxiety disorders was slightly concentrated among nations with higher SDI, yet inequities narrowed over time, whereas depressive disorders paradoxically showed a greater concentration among advantaged groups and these inequities remained largely unchanged. During the COVID‐19 pandemic, incidence rates of both disorders rose markedly, with the sharpest relative increases observed among younger populations and in regions with higher SDI, including Europe, North America, and Latin America. After adjustment for potential confounders, both rising unemployment and greater government stringency remained independently and significantly associated with larger ASIR increases for anxiety and depressive disorders, with the strongest effects observed among females and younger age groups.

Our findings on age‐ and sex‐specific patterns of anxiety and depressive disorders align with previous studies (Beesdo et al. 2010; Patwardhan et al. 2024). This agreement with prior evidence strengthens the reliability of our estimates and highlights the enduring demographic disparities in mental health burden. The observed pandemic‐related increases are consistent with emerging population‐based evidence, as systematic reviews and meta‐analyses have shown marked rises in anxiety and depressive disorders during COVID‐19, particularly among younger individuals affected by social isolation, educational disruption, and uncertainty (Delpino et al. 2022; Madigan et al. 2023; Silva et al. 2023). This study builds on this evidence by quantifying global incidence trends. We confirm that the pandemic surge was not confined to a single region but occurred across diverse socioeconomic contexts, thereby extending earlier survey‐based studies that were largely limited to high‐income countries.

Beyond demographic patterns, our results add to ongoing debates regarding socioeconomic inequalities in mental health. Prior comparative evidence suggests that the burden of anxiety disorders is higher in high‐income countries, likely driven by greater diagnostic capacity and awareness (Ruscio et al. 2017). In contrast, depressive disorders tend to contribute a larger burden in low‐ and middle‐income countries, where structural risks such as limited treatment availability and stigma exacerbate vulnerability (Moitra et al. 2022; Javed et al. 2021). Our observation that depression‐related inequalities narrowed over time, whereas anxiety disorders became more prominent in disadvantaged settings, likely reflects distinct mechanisms across SDI levels. High‐SDI countries experienced larger short‐term increases during the pandemic, probably due to greater diagnostic capacity, heightened awareness, and stricter containment measures that intensified psychological stress. In contrast, lower‐SDI regions continued to bear a higher long‐term depressive burden, which may be driven by limited mental health resources, persistent stigma, and lower treatment coverage. These patterns were consistent with population‐based studies from high‐income settings that reported sharp deteriorations in mental health during periods of intense infection waves and stringent public health restrictions (Pierce et al. 2020; Ettman et al. 2020; Chan et al. 2024).

The associations between socioeconomic determinants and mental disorders identified in our study are consistent with earlier evidence. Previous research has repeatedly demonstrated that unemployment (Ringlein et al. 2024; Freund et al. 2025; Martin et al. 2025) and stringent government responses (Salanti et al. 2022; Aknin et al. 2022; Liu et al. 2024) were associated with elevated risks of anxiety and depression, with disproportionately stronger effects among women and younger individuals. Most of these studies, however, were conducted in specific populations, predominantly in high‐income countries, and thus provide limited insights into socioeconomic variations across diverse global contexts. Our analysis extends this evidence by providing a global perspective, demonstrating that economic shocks and containment policies were systematically associated with increases in ASIRs of anxiety and depressive disorders, even after accounting for the severity of the COVID‐19 epidemic and SDI levels. These findings highlight the robustness of socioeconomic determinants as factors associated with mental health burden during crises and underscore the importance of integrating macro‐level socioeconomic resilience into preparedness strategies.

Several mechanisms may explain the associations observed between socioeconomic determinants and the burden of anxiety and depressive disorders. Unemployment and wider economic disruption constitute established stressors that elevate psychiatric risk through heightened financial insecurity, loss of social status, and erosion of daily routine. Such shocks not only increased financial stress to individuals but also undermined social protection systems in many regions, thereby amplifying mental health risks (Witteveen and Velthorst 2020). Government containment policies, though crucial for mitigating viral transmission, represented an unprecedented social intervention at scale. Restrictions on mobility, closure of schools and workplaces, and prolonged lockdowns disrupted established social and economic routines (Brooks et al. 2020). These disruptions affected community networks, restricted access to health and social services, and increased overall uncertainty, which in turn might contribute to elevated rates of anxiety and depression across the population.

The differential effects observed across sex and age likely reflect variations in vulnerability and resilience. Previous data indicate that women assumed substantially increased caregiving and household responsibilities, which were strongly associated with elevated depression, anxiety, and post‐traumatic stress disorders (Eugene et al. 2025). Pandemic‐related home and work role imbalances contributed to greater burnout and work–family conflict among women compared to men (Stefanova et al. 2021). Younger individuals were disproportionately affected by pandemic‐related economic and social disruptions. Cross‐national data indicate that employment and income losses during COVID‐19 significantly compromised the psychological well‐being of young adults (Li et al. 2023). Among adolescents, experiences of loneliness and isolation were strongly linked to increased symptoms of anxiety and depression (Temple et al. 2022).

Our findings have several implications for policy and practice. Mental health care should be integrated into emergency preparedness, rather than added after crises emerge. Women and younger people carried the heaviest burden, pointing to the value of school‐based programs, workplace support, and services for caregivers. Protecting mental health also requires cushioning the economic shock of unemployment with timely financial and social support. When restrictive public health measures are needed, their potential psychological costs should be weighed carefully, and mitigation strategies put in place.

This study has several strengths. It is, to our knowledge, the first to provide a global assessment of age, sex, and socioeconomic disparities in the incidence of anxiety and depressive disorders during the COVID‐19 pandemic, drawing on GBD 2021 estimates across 204 countries and territories. By linking incidence trends to socioeconomic indicators such as unemployment and government stringency, the study offers new insights into macro‐level drivers of mental health burden. However, several limitations should be acknowledged. First, the ecological study design cannot exclude ecological fallacy and cannot establish causality due to its cross‐sectional nature. Second, the data used in this study also have inherent limitations. Although the GSI is a standardized and widely used composite indicator, it may not fully capture the heterogeneity of government responses across countries. Variations in enforcement intensity or specific policy domains could introduce measurement bias. Moreover, data from different sources may vary in collection and reporting methodologies. GBD estimates are periodically revised as new data sources and methodological improvements become available, which may lead to minor changes in absolute values in future releases. In addition, this analysis focused on the first year of the COVID‐19 pandemic and therefore may not fully reflect long‐term trends in the global burden of anxiety and depressive disorders. Third, the measurement of government response stringency through the GSI may not capture all relevant policy complexities. Fourth, the statistical model may not account for all potential confounding factors, such as cultural and health care system differences.

## 5 Conclusions

The COVID‐19 pandemic was associated with unprecedented increases in the global incidence of anxiety and depressive disorders, with the heaviest burden observed among women, younger individuals, and populations in high‐SDI regions. Our analysis also shows that rising unemployment and more stringent government responses were linked to higher incidence rates, even after accounting for epidemic severity. These findings indicate that economic shocks and containment measures, while essential for infection control, may have contributed substantially to the mental health burden. Addressing these impacts will require integrating mental health considerations into emergency preparedness, providing targeted support for vulnerable groups, and strengthening economic protections and access to mental health services as part of broader public health strategies in future crises.

## Author Contributions

**Chen Zhao**: conceptualization, formal analysis, funding acquisition, writing – original draft. **Xīn Gào**: methodology, data curation, investigation, formal analysis, visualization, writing – review and editing. **Mengdi Zhang**: data curation, project administration. **Weihua Yue**: conceptualization, supervision, funding acquisition, writing – review and editing. **Xiao Zhang**: conceptualization, supervision, funding acquisition, writing – review and editing.

## Funding

The current study was supported by the Fundamental Research Funds for the Central Universities (Peking University Clinical Scientist Training Program, BMU2024PYJH020, to X.Z.), Beijing Natural Science Foundation (7254447, to C.Z.), the Postdoctoral Fellowship Program of China Postdoctoral Science Foundation (GZC20240064, to X.G.), National Natural Science Foundation of China (82441005, 82330042, to W.Y.), and National Key R&D Program of China (2023YFE0119400, to W.Y.).

## Ethics Statement

The authors have nothing to report.

## Conflicts of Interest

The authors declare no conflicts of interest.

## Supporting information




**Supplementary Material**: brb371132‐Supp‐0001‐Mat.docx

## Data Availability

The data that support the findings of this study are available on request from the corresponding author. The data are not publicly available due to privacy or ethical restrictions.
