# Supplementary material for: Association of Unemployment and Government Stringency With the Increased Burden of Anxiety and Depression Amid the Public Health Emergency: A Global Perspective
Source: Brain Behav. 2025 Dec 10;15(12):e71132. doi: 10.1002/brb3.71132 (PMC12696339; doi:10.1002/brb3.71132)
Supplement: Supplementary file 1 — Supplementary Material: brb371132‐Supp‐0001‐Mat.docx [file BRB3-15-e71132-s001.docx]

**Supplementary Materials**

**Title: Association of Unemployment and Government Stringency with the Increased Burden of Anxiety and Depression amid the Public Health Emergency: A Global Perspective**

**Supplementary Tables**

| **Supplementary table A.1** Countries and territories removed from analysis | |
| --- | --- |
| Locations | Socio-demographic index |
| Guinea-Bissau | 34.78 |
| Côte d'Ivoire | 42.59 |
| Comoros | 47.20 |
| Sao Tome and Principe | 49.79 |
| Kiribati | 52.35 |
| Democratic People's Republic of Korea | 56.82 |
| Marshall Islands | 56.88 |
| Tuvalu | 57.17 |
| Micronesia | 58.45 |
| Samoa | 59.01 |
| Nauru | 61.93 |
| Saint Vincent and the Grenadines | 63.29 |
| Equatorial Guinea | 65.21 |
| Grenada | 66.51 |
| Saint Lucia | 66.97 |
| Tokelau | 68.29 |
| Armenia | 69.83 |
| American Samoa | 72.08 |
| Niue | 72.27 |
| Seychelles | 72.67 |
| Dominica | 74.44 |
| Antigua and Barbuda | 74.63 |
| North Macedonia | 74.78 |
| Saint Kitts and Nevis | 75.16 |
| Palau | 75.28 |
| Northern Mariana Islands | 76.94 |
| Cook Islands | 77.47 |
| Montenegro | 79.26 |
| Bermuda | 81.92 |
| Greenland | 82.44 |
| Andorra | 86.74 |
| Taiwan (Province of China) | 87.12 |
| San Marino | 88.69 |
| Monaco | 90.67 |

| **Supplementary table A.2** Government policy responses to COVID-19 by SDI levels, 2020 | | | | | |
| --- | --- | --- | --- | --- | --- |
|  | **Low SDI** | **Low-middle SDI** | **Middle SDI** | **High-middle SDI** | **High SDI** |
|  | **n (%)** | **n (%)** | **n (%)** | **n (%)** | **n (%)** |
| **COVID-19 testing policies** |  |  |  |  |  |
| 0. No testing policy | 3 (9.4) | 0 (0) | 0 (0) | 0 (0) | 0 (0) |
| 1. Testing only for those who both (a) have symptoms AND (b) meet specific criteria | 20 (62.5) | 21 (60) | 16 (48.5) | 9 (24.3) | 4 (9.8) |
| 2. Testing of anyone showing COVID-19 symptoms | 8 (25) | 12 (34.3) | 14 (42.4) | 14 (37.8) | 23 (56.1) |
| 3. Open public testing | 1 (3.1) | 2 (5.7) | 3 (9.1) | 14 (37.8) | 14 (34.1) |
| **COVID-19 contact tracing policies** |  |  |  |  |  |
| 0. No tracing | 6 (18.8) | 4 (11.4) | 2 (6.1) | 1 (2.7) | 3 (7.3) |
| 1. Limited tracing | 12 (37.5) | 19 (54.3) | 13 (39.4) | 9 (24.3) | 9 (22) |
| 2. Comprehensive tracing | 14 (43.8) | 12 (34.3) | 18 (54.5) | 27 (73) | 29 (70.7) |
| **Face covering policies** |  |  |  |  |  |
| 0. No policy | 10 (31.2) | 6 (17.1) | 10 (30.3) | 7 (18.9) | 14 (34.1) |
| 1. Recommended | 1 (3.1) | 2 (5.7) | 1 (3) | 1 (2.7) | 3 (7.3) |
| 2. Required in some specified shared/public spaces outside the home with other people present, or some situations when social distancing not possible | 2 (6.2) | 1 (2.9) | 2 (6.1) | 13 (35.1) | 9 (22) |
| 3. Required in all shared/public spaces outside the home with other people present or all situations when social distancing not possible | 15 (46.9) | 18 (51.4) | 13 (39.4) | 12 (32.4) | 12 (29.3) |
| 4. Required outside the home at all times regardless of location or presence of other people | 4 (12.5) | 8 (22.9) | 7 (21.2) | 4 (10.8) | 3 (7.3) |
| **Public events cancellation** |  |  |  |  |  |
| 0. No measures | 9 (28.1) | 6 (17.1) | 2 (6.1) | 2 (5.4) | 4 (9.8) |
| 1. Recommended cancellations | 6 (18.8) | 3 (8.6) | 4 (12.1) | 7 (18.9) | 11 (26.8) |
| 2. Required cancellations | 17 (53.1) | 26 (74.3) | 27 (81.8) | 28 (75.7) | 26 (63.4) |
| **Public gathering cancellation** |  |  |  |  |  |
| 0. No restrictions | 10 (31.2) | 6 (17.1) | 3 (9.1) | 7 (18.9) | 6 (14.6) |
| 1. Restrictions on very large gatherings (the limit is above 1000 people) | 0 (0) | 0 (0) | 0 (0) | 0 (0) | 0 (0) |
| 2. Restrictions on gatherings between 100 to 1000 people | 3 (9.4) | 1 (2.9) | 3 (9.1) | 3 (8.1) | 2 (4.9) |
| 3. Restrictions on gatherings between 10 to 100 people | 13 (40.6) | 15 (42.9) | 13 (39.4) | 7 (18.9) | 10 (24.4) |
| 4. Restrictions on gatherings of less than 10 people | 6 (18.8) | 13 (37.1) | 14 (42.4) | 20 (54.1) | 23 (56.1) |
| **Public transport restrictions** |  |  |  |  |  |
| 0. No measures | 23 (71.9) | 22 (62.9) | 17 (51.5) | 24 (64.9) | 28 (68.3) |
| 1. Recommended closing or reduce volume | 6 (18.8) | 8 (22.9) | 8 (24.2) | 7 (18.9) | 11 (26.8) |
| 2. Required closing or prohibit most citizens from using it | 3 (9.4) | 5 (14.3) | 8 (24.2) | 6 (16.2) | 2 (4.9) |
| **School closures** |  |  |  |  |  |
| 0. No measures | 4 (12.5) | 4 (11.4) | 0 (0) | 2 (5.4) | 2 (4.9) |
| 1. Recommended | 5 (15.6) | 1 (2.9) | 5 (15.2) | 5 (13.5) | 15 (36.6) |
| 2. Require closing only at some levels | 6 (18.8) | 8 (22.9) | 6 (18.2) | 10 (27) | 13 (31.7) |
| 3. require closing (or work from home) for all-but-essential workplaces (eg grocery stores, doctors) | 17 (53.1) | 22 (62.9) | 22 (66.7) | 20 (54.1) | 11 (26.8) |
| **Workplace closures** |  |  |  |  |  |
| 0. No measures | 15 (46.9) | 5 (14.3) | 5 (15.2) | 7 (18.9) | 6 (14.6) |
| 1. Recommended | 2 (6.2) | 7 (20) | 3 (9.1) | 5 (13.5) | 12 (29.3) |
| 2. Require closing (or work from home) for some sectors or categories of workers | 14 (43.8) | 19 (54.3) | 22 (66.7) | 23 (62.2) | 22 (53.7) |
| 3. Required at all levels | 1 (3.1) | 4 (11.4) | 3 (9.1) | 2 (5.4) | 1 (2.4) |
| **Stay-at-home restrictions** |  |  |  |  |  |
| 0. No measures | 18 (56.2) | 15 (42.9) | 5 (15.2) | 16 (43.2) | 23 (56.1) |
| 1. Recommended not to leave the house | 7 (21.9) | 4 (11.4) | 7 (21.2) | 9 (24.3) | 14 (34.1) |
| 2. Required to not leave the house with exceptions for daily exercise, grocery shopping, and ‘essential’ trips | 7 (21.9) | 15 (42.9) | 21 (63.6) | 10 (27) | 4 (9.8) |
| 3. Required to not leave the house with minimal exceptions | 0 (0) | 1 (2.9) | 0 (0) | 2 (5.4) | 0 (0) |

SDI=socio-demographic index.

Note: COVID-19 testing policies, COVID-19 contact tracing policies, face covering policies, public events cancellation, public gathering cancellation, public transport restrictions, school closures, and workplace closures were the mode values throughout 2020.

| **Supplementary table A.3** Association between increase in unemployment rate and increase in incidence of anxiety disorders and depressive disorders | | | | | |
| --- | --- | --- | --- | --- | --- |
|  | **Anxiety disorders** | |  | **Depressive disorders** | |
| **Indicators** | **Coefficient** | **p** |  | **Coefficient** | **p** |
| **Overall population** |  |  |  |  |  |
| COVID-19 incidence rate in 2020 | 5.00 (4.05 to 5.96) | <0.0001 |  | 3.73 (2.72 to 4.75) | <0.0001 |
| Socio-demographic index in 2020 | 1.72 (-0.03 to 3.47) | 0.0561 |  | 2.59 (0.72 to 4.45) | 0.0072 |
| Median age in 2020 | 2.13 (0.27 to 3.99) | 0.0259 |  | 1.30 (-0.68 to 3.27) | 0.1993 |
| Female percentage in 2020 | 1.21 (0.32 to 2.10) | 0.0082 |  | 1.04 (0.09 to 1.98) | 0.0330 |
| Unemployment rate change, 2019-2020 | 1.43 (0.56 to 2.29) | 0.0015 |  | 1.70 (0.78 to 2.62) | 0.0004 |
| Median stringency index in 2020 | 1.14 (0.24 to 2.04) | 0.0142 |  | 1.29 (0.33 to 2.24) | 0.0092 |
| **Female** |  |  |  |  |  |
| COVID-19 incidence rate in 2020 | 5.23 (4.23 to 6.24) | <0.0001 |  | 3.81 (2.73 to 4.89) | <0.0001 |
| Socio-demographic index in 2020 | 1.84 (-0.00 to 3.69) | 0.0520 |  | 2.56 (0.58 to 4.54) | 0.0122 |
| Median age in 2020 | 2.21 (0.25 to 4.16) | 0.0282 |  | 1.49 (-0.61 to 3.58) | 0.1668 |
| Female percentage in 2020 | 1.32 (0.39 to 2.26) | 0.0061 |  | 1.11 (0.10 to 2.11) | 0.0321 |
| Unemployment rate change, 2019-2020 | 1.51 (0.60 to 2.42) | 0.0014 |  | 1.76 (0.78 to 2.73) | 0.0005 |
| Median stringency index in 2020 | 1.19 (0.24 to 2.14) | 0.0149 |  | 1.38 (0.36 to 2.39) | 0.0086 |
| **Male** |  |  |  |  |  |
| COVID-19 incidence rate in 2020 | 4.70 (3.81 to 5.59) | <0.0001 |  | 3.70 (2.77 to 4.64) | <0.0001 |
| Socio-demographic index in 2020 | 1.57 (-0.07 to 3.22) | 0.0620 |  | 2.65 (0.93 to 4.36) | 0.0029 |
| Median age in 2020 | 1.97 (0.23 to 3.71) | 0.0277 |  | 1.13 (-0.69 to 2.95) | 0.2244 |
| Female percentage in 2020 | 1.10 (0.27 to 1.93) | 0.0106 |  | 1.00 (0.13 to 1.87) | 0.0249 |
| Unemployment rate change, 2019-2020 | 1.33 (0.52 to 2.14) | 0.0016 |  | 1.58 (0.73 to 2.42) | 0.0004 |
| Median stringency index in 2020 | 1.07 (0.23 to 1.91) | 0.0139 |  | 1.11 (0.22 to 1.99) | 0.0150 |
| **Children and adolescents (≤19 years)** |  |  |  |  |  |
| COVID-19 incidence rate in 2020 | 7.03 (5.70 to 8.37) | <0.0001 |  | 8.61 (6.63 to 10.60) | <0.0001 |
| Socio-demographic index in 2020 | 2.23 (-0.22 to 4.67) | 0.0768 |  | 2.91 (-0.74 to 6.56) | 0.1198 |
| Median age in 2020 | 2.91 (0.32 to 5.51) | 0.0292 |  | 3.70 (-0.17 to 7.57) | 0.0628 |
| Female percentage in 2020 | 1.87 (0.63 to 3.11) | 0.0037 |  | 2.27 (0.42 to 4.12) | 0.0172 |
| Unemployment rate change, 2019-2020 | 2.21 (1.00 to 3.42) | 0.0005 |  | 3.11 (1.31 to 4.91) | 0.0009 |
| Median stringency index in 2020 | 1.46 (0.21 to 2.72) | 0.0238 |  | 2.12 (0.25 to 4.00) | 0.0278 |
| **Working age adults (20 to ≤64 years)** |  |  |  |  |  |
| COVID-19 incidence rate in 2020 | 4.14 (3.37 to 4.91) | <0.0001 |  | 3.95 (2.97 to 4.93) | <0.0001 |
| Socio-demographic index in 2020 | 1.30 (-0.12 to 2.72) | 0.0743 |  | 2.34 (0.54 to 4.14) | 0.0117 |
| Median age in 2020 | 1.76 (0.26 to 3.27) | 0.0230 |  | 1.43 (-0.48 to 3.34) | 0.1442 |
| Female percentage in 2020 | 1.06 (0.34 to 1.78) | 0.0045 |  | 1.22 (0.30 to 2.13) | 0.0098 |
| Unemployment rate change, 2019-2020 | 1.24 (0.54 to 1.94) | 0.0007 |  | 1.81 (0.92 to 2.70) | <0.0001 |
| Median stringency index in 2020 | 1.02 (0.29 to 1.75) | 0.0071 |  | 1.34 (0.41 to 2.26) | 0.0052 |
| **Elderly population (≥65 years)** |  |  |  |  |  |
| COVID-19 incidence rate in 2020 | 1.51 (1.25 to 1.78) | <0.0001 |  | 0.69 (0.32 to 1.06) | 0.0003 |
| Socio-demographic index in 2020 | 0.35 (-0.14 to 0.84) | 0.1673 |  | 0.72 (0.04 to 1.40) | 0.0392 |
| Median age in 2020 | 0.57 (0.05 to 1.09) | 0.0325 |  | -0.05 (-0.77 to 0.67) | 0.8851 |
| Female percentage in 2020 | 0.34 (0.10 to 0.59) | 0.0074 |  | 0.31 (-0.03 to 0.65) | 0.0778 |
| Unemployment rate change, 2019-2020 | 0.40 (0.16 to 0.64) | 0.0013 |  | 0.44 (0.10 to 0.77) | 0.0116 |
| Median stringency index in 2020 | 0.33 (0.08 to 0.59) | 0.0100 |  | 0.17 (-0.18 to 0.52) | 0.3418 |

| **Supplementary table A.3** Association between government stringency index and increase in incidence of anxiety disorders and depressive disorders | | | | | |
| --- | --- | --- | --- | --- | --- |
|  | **Anxiety disorders** | |  | **Depressive disorders** | |
| **Indicators** | **Coefficient** | **p** |  | **Coefficient** | **p** |
| **Overall population** |  |  |  |  |  |
| Median stringency index in 2020 | 1.08 (0.19 to 1.98) | 0.0192 |  | 1.34 (0.41 to 2.28) | 0.0053 |
| COVID-19 incidence rate in 2020 | 4.98 (3.98 to 5.97) | <0.0001 |  | 3.91 (2.88 to 4.95) | <0.0001 |
| Socio-demographic index in 2020 | 2.85 (1.45 to 4.26) | 0.0001 |  | 2.56 (1.10 to 4.02) | 0.0007 |
| Health workforce | -0.37 (-2.16 to 1.42) | 0.6845 |  | -0.33 (-2.19 to 1.53) | 0.7283 |
| Health expenditure | 1.48 (-0.11 to 3.07) | 0.0698 |  | 2.25 (0.60 to 3.91) | 0.0082 |
| **Female** |  |  |  |  |  |
| Median stringency index in 2020 | 1.14 (0.19 to 2.08) | 0.0197 |  | 1.44 (0.45 to 2.42) | 0.0050 |
| COVID-19 incidence rate in 2020 | 5.22 (4.17 to 6.27) | <0.0001 |  | 3.99 (2.89 to 5.09) | <0.0001 |
| Socio-demographic index in 2020 | 2.90 (1.42 to 4.38) | <0.0001 |  | 2.61 (1.06 to 4.15) | 0.0011 |
| Health workforce | -0.32 (-2.21 to 1.56) | 0.7387 |  | -0.28 (-2.25 to 1.70) | 0.7850 |
| Health expenditure | 1.63 (-0.05 to 3.31) | 0.0583 |  | 2.32 (0.57 to 4.07) | 0.0103 |
| **Male** |  |  |  |  |  |
| Median stringency index in 2020 | 1.02 (0.17 to 1.86) | 0.019 |  | 1.14 (0.28 to 2.00) | 0.0101 |
| COVID-19 incidence rate in 2020 | 4.66 (3.73 to 5.59) | <0.0001 |  | 3.86 (2.91 to 4.82) | <0.0001 |
| Socio-demographic index in 2020 | 2.75 (1.44 to 4.06) | <0.0001 |  | 2.63 (1.28 to 3.97) | 0.0002 |
| Health workforce | -0.45 (-2.13 to 1.22) | 0.5985 |  | -0.43 (-2.14 to 1.29) | 0.6253 |
| Health expenditure | 1.31 (-0.18 to 2.80) | 0.0868 |  | 2.11 (0.58 to 3.64) | 0.0074 |
| **Children and adolescents (≤19 years)** | |  |  |  |  |
| Median stringency index in 2020 | 1.43 (0.16 to 2.70) | 0.0288 |  | 2.12 (0.27 to 3.97) | 0.0259 |
| COVID-19 incidence rate in 2020 | 6.91 (5.50 to 8.32) | <0.0001 |  | 8.57 (6.52 to 10.62) | <0.0001 |
| Socio-demographic index in 2020 | 3.95 (1.96 to 5.94) | 0.0001 |  | 4.68 (1.79 to 7.57) | 0.0018 |
| Health workforce | -0.53 (-3.06 to 2.01) | 0.6849 |  | -0.60 (-4.29 to 3.09) | 0.7518 |
| Health expenditure | 1.74 (-0.51 to 4.00) | 0.1312 |  | 2.87 (-0.41 to 6.15) | 0.0879 |
| **Working age adults (20 to ≤64 years)** | |  |  |  |  |
| Median stringency index in 2020 | 0.99 (0.25 to 1.72) | 0.0096 |  | 1.45 (0.53 to 2.36) | 0.0024 |
| COVID-19 incidence rate in 2020 | 4.06 (3.25 to 4.88) | <0.0001 |  | 4.04 (3.03 to 5.06) | <0.0001 |
| Socio-demographic index in 2020 | 2.35 (1.20 to 3.50) | 0.0001 |  | 2.55 (1.11 to 3.98) | 0.0006 |
| Health workforce | -0.33 (-1.80 to 1.14) | 0.6567 |  | -0.12 (-1.95 to 1.71) | 0.8976 |
| Health expenditure | 1.06 (-0.25 to 2.36) | 0.1148 |  | 1.76 (0.14 to 3.39) | 0.0350 |
| **Elderly population (≥65 years)** |  |  |  |  |  |
| Median stringency index in 2020 | 0.31 (0.06 to 0.56) | 0.0167 |  | 0.27 (-0.06 to 0.60) | 0.1114 |
| COVID-19 incidence rate in 2020 | 1.42 (1.14 to 1.69) | <0.0001 |  | 0.73 (0.36 to 1.09) | 0.0001 |
| Socio-demographic index in 2020 | 0.94 (0.55 to 1.34) | <0.0001 |  | 0.48 (-0.04 to 0.99) | 0.0696 |
| Health workforce | -0.38 (-0.88 to 0.12) | 0.1384 |  | 0.08 (-0.57 to 0.74) | 0.8022 |
| Health expenditure | 0.23 (-0.22 to 0.67) | 0.3181 |  | 0.21 (-0.37 to 0.79) | 0.4835 |

| **Supplementary table A.4** Sensitivity analyses for the associations of potential influence indicators with percentage change in incidence rate of anxiety and depressive disorders by sex | | | | | | |
| --- | --- | --- | --- | --- | --- | --- |
|  |  | **Female** | |  | **Male** | |
| **Dependent variables** | **Z-score normalized indicators** | **ꞵ (95% CI)** | **p values** |  | **ꞵ (95% CI)** | **p values** |
| Increase in anxiety disorders  incidence rate | COVID-19 incidence rate in 2020 | 4.95 (3.82 to 6.08) | <0.0001 |  | 4.51 (3.50 to 5.52) | <0.0001 |
|  | Socio-demographic index in 2020 | 3.34 (2.17 to 4.52) | <0.0001 |  | 2.96 (1.90 to 4.01) | <0.0001 |
|  | GDP per capita change,2019-2020 | -0.57  (-1.59 to 0.46) | 0.2809 |  | -0.50  (-1.42 to 0.42) | 0.2852 |
|  | CPI annual change, 2019-2020 | -0.47 (-1.48 to 0.55) | 0.3673 |  | -0.38 (-1.29 to 0.53) | 0.4113 |
|  | Unemployment rate change, 2019-2020 | 1.47 (0.42 to 2.52) | 0.0071 |  | 1.05 (0.11 to 2.00) | 0.0307 |
|  | Economic support index in 2020 | 1.11 (-0.04 to 2.25) | 0.0613 |  | 0.93 (-0.10 to 1.96) | 0.0805 |
|  | Stringency index in 2020 | 1.15 (0.03 to 2.26) | 0.0452 |  | 1.11 (0.11 to 2.10) | 0.0304 |
| Increase in depressive disorders  incidence rate | COVID-19 incidence rate in 2020 | 3.74 (2.54 to 4.93) | <0.0001 |  | 3.62 (2.58 to 4.65) | <0.0001 |
|  | Socio-demographic index in 2020 | 3.67 (2.43 to 4.91) | <0.0001 |  | 3.39 (2.31 to 4.46) | <0.0001 |
|  | GDP per capita change,2019-2020 | -0.53 (-1.61 to 0.55) | 0.3404 |  | -0.42 (-1.37 to 0.52) | 0.3780 |
|  | CPI annual change, 2019-2020 | -0.38 (-1.45 to 0.69) | 0.4844 |  | -0.28 (-1.21 to 0.65) | 0.5553 |
|  | Unemployment rate change, 2019-2020 | 1.74 (0.64 to 2.85) | 0.0024 |  | 1.38 (0.42 to 2.35) | 0.0058 |
|  | Economic support index in 2020 | 1.02 (-0.19 to 2.23) | 0.1008 |  | 1.01 (-0.04 to 2.07) | 0.0624 |
|  | Stringency index in 2020 | 1.28 (0.11 to 2.45) | 0.0339 |  | 1.14 (0.12 to 2.15) | 0.0299 |

CPI=consumer price index; GDP=gross domestic product.

| **Supplementary table A.5** Sensitivity analyses for the associations between percentage change in incidence rate of anxiety disorders and goverment restrictions in response to the COVID-19 pandemic by age group | | |
| --- | --- | --- |
| **Z-score normalized indicators** | **ꞵ (95% CI)** | **p values** |
| **All ages** |  |  |
| COVID-19 incidence rate in 2020 | 4.68 (3.57 to 5.78) | <0.0001 |
| Socio-demographic index in 2020 | 3.93 (2.79 to 5.07) | <0.0001 |
| COVID-19 contact tracing policies | -0.88 (-1.90 to 0.13) | 0.0890 |
| Face covering policies | 0.44 (-0.60 to 1.48) | 0.4085 |
| Public events cancellation | 0.38 (-0.91 to 1.68) | 0.5633 |
| Public gathering cancellation | 0.55 (-0.71 to 1.81) | 0.3950 |
| Stay-at-home restrictions | 1.11 (-0.01 to 2.23) | 0.0548 |
| **Children and adolescents (≤19 years)** | |  |
| COVID-19 incidence rate in 2020 | 6.58 (5.02 to 8.15) | <0.0001 |
| Socio-demographic index in 2020 | 5.21 (3.60 to 6.83) | <0.0001 |
| COVID-19 contact tracing policies | -1.20 (-2.63 to 0.23) | 0.1027 |
| Face covering policies | 0.66 (-0.82 to 2.13) | 0.3840 |
| Public events cancellation | 0.41 (-1.43 to 2.25) | 0.6644 |
| Public gathering cancellation | 0.74 (-1.04 to 2.52) | 0.4166 |
| Stay-at-home restrictions | 1.61 (0.02 to 3.20) | 0.0495 |
| **Working age adults (20 to ≤64 years)** | |  |
| COVID-19 incidence rate in 2020 | 3.84 (2.94 to 4.74) | <0.0001 |
| Socio-demographic index in 2020 | 3.09 (2.16 to 4.03) | <0.0001 |
| COVID-19 contact tracing policies | -0.70 (-1.52 to 0.13) | 0.0996 |
| Face covering policies | 0.34 (-0.52 to 1.21) | 0.4400 |
| Public events cancellation | 0.24 (-0.85 to 1.32) | 0.6687 |
| Public gathering cancellation | 0.48 (-0.55 to 1.51) | 0.3619 |
| Workplace closures | 0.10 (-0.85 to 1.05) | 0.8423 |
| Stay-at-home restrictions | 1.12 (0.19 to 2.04) | 0.0194 |
| **Elderly population (≥65 years)** |  |  |
| COVID-19 incidence rate in 2020 | 1.39 (1.08 to 1.70) | <0.0001 |
| Socio-demographic index in 2020 | 0.91 (0.60 to 1.22) | <0.0001 |
| COVID-19 contact tracing policies | -0.23 (-0.51 to 0.04) | 0.1021 |
| Face covering policies | 0.14 (-0.15 to 0.43) | 0.3545 |
| Public events cancellation | 0.11 (-0.26 to 0.48) | 0.5676 |
| Public gathering cancellation | 0.12 (-0.23 to 0.47) | 0.5049 |
| Public transport restrictions | 0.04 (-0.26 to 0.33) | 0.8169 |
| School closures | 0.04 (-0.31 to 0.39) | 0.8164 |
| Stay-at-home restrictions | 0.40 (0.07 to 0.73) | 0.0181 |

| **Supplementary table A.6** Sensitivity analyses for the associations between percentage change in incidence rate of depressive disorders and goverment restrictions in response to the COVID-19 pandemic by age group | | |
| --- | --- | --- |
| **Z-score normalized indicators** | **ꞵ (95% CI)** | **p values** |
| **All ages** |  |  |
| COVID-19 incidence rate in 2020 | 3.57 (2.41 to 4.73) | <0.0001 |
| Socio-demographic index in 2020 | 4.25 (3.04 to 5.45) | <0.0001 |
| COVID-19 contact tracing policies | -0.76 (-1.82 to 0.30) | 0.1617 |
| Face covering policies | 0.51 (-0.60 to 1.63) | 0.3679 |
| Public events cancellation | 0.35 (-1.05 to 1.74) | 0.6276 |
| Public gathering cancellation | 0.52 (-0.81 to 1.85) | 0.4451 |
| Public transport restrictions | 0.19 (-0.94 to 1.33) | 0.7404 |
| Workplace closures | 0.10 (-1.14 to 1.34) | 0.8730 |
| Stay-at-home restrictions | 1.40 (0.17 to 2.63) | 0.0273 |
| **Children and adolescents (≤19 years)** |  |  |
| COVID-19 incidence rate in 2020 | 8.12 (5.79 to 10.46) | <0.0001 |
| Socio-demographic index in 2020 | 7.11 (4.50 to 9.72) | <0.0001 |
| COVID-19 testing policies | -0.94 (-3.40 to 1.51) | 0.4527 |
| COVID-19 contact tracing policies | -1.30 (-3.45 to 0.86) | 0.2398 |
| Face covering policies | 0.87 (-1.37 to 3.11) | 0.4478 |
| Public events cancellation | 0.65 (-2.24 to 3.53) | 0.6609 |
| Public gathering cancellation | 1.22 (-1.45 to 3.90) | 0.3714 |
| Public transport restrictions | 0.45 (-1.83 to 2.74) | 0.6988 |
| Workplace closures | 0.06 (-2.43 to 2.56) | 0.9622 |
| Stay-at-home restrictions | 2.23 (-0.26 to 4.71) | 0.0819 |
| **Working age adults (20 to ≤64 years)** |  |  |
| COVID-19 incidence rate in 2020 | 3.73 (2.59 to 4.88) | <0.0001 |
| Socio-demographic index in 2020 | 4.01 (2.82 to 5.20) | <0.0001 |
| COVID-19 contact tracing policies | -0.67 (-1.71 to 0.38) | 0.2121 |
| Face covering policies | 0.46 (-0.63 to 1.56) | 0.4108 |
| Public events cancellation | 0.29 (-1.09 to 1.66) | 0.6822 |
| Public gathering cancellation | 0.55 (-0.77 to 1.86) | 0.4166 |
| Public transport restrictions | 0.13 (-0.99 to 1.25) | 0.8262 |
| Workplace closures | 0.19 (-1.03 to 1.41) | 0.7609 |
| Stay-at-home restrictions | 1.49 (0.28 to 2.70) | 0.0170 |
| **Elderly population (≥65 years)** |  |  |
| COVID-19 incidence rate in 2020 | 0.70 (0.28 to 1.12) | 0.0014 |
| Socio-demographic index in 2020 | 0.82 (0.42 to 1.22) | <0.0001 |
| School closures | 0.37 (-0.02 to 0.77) | 0.0680 |

**Supplementary Figures**

**
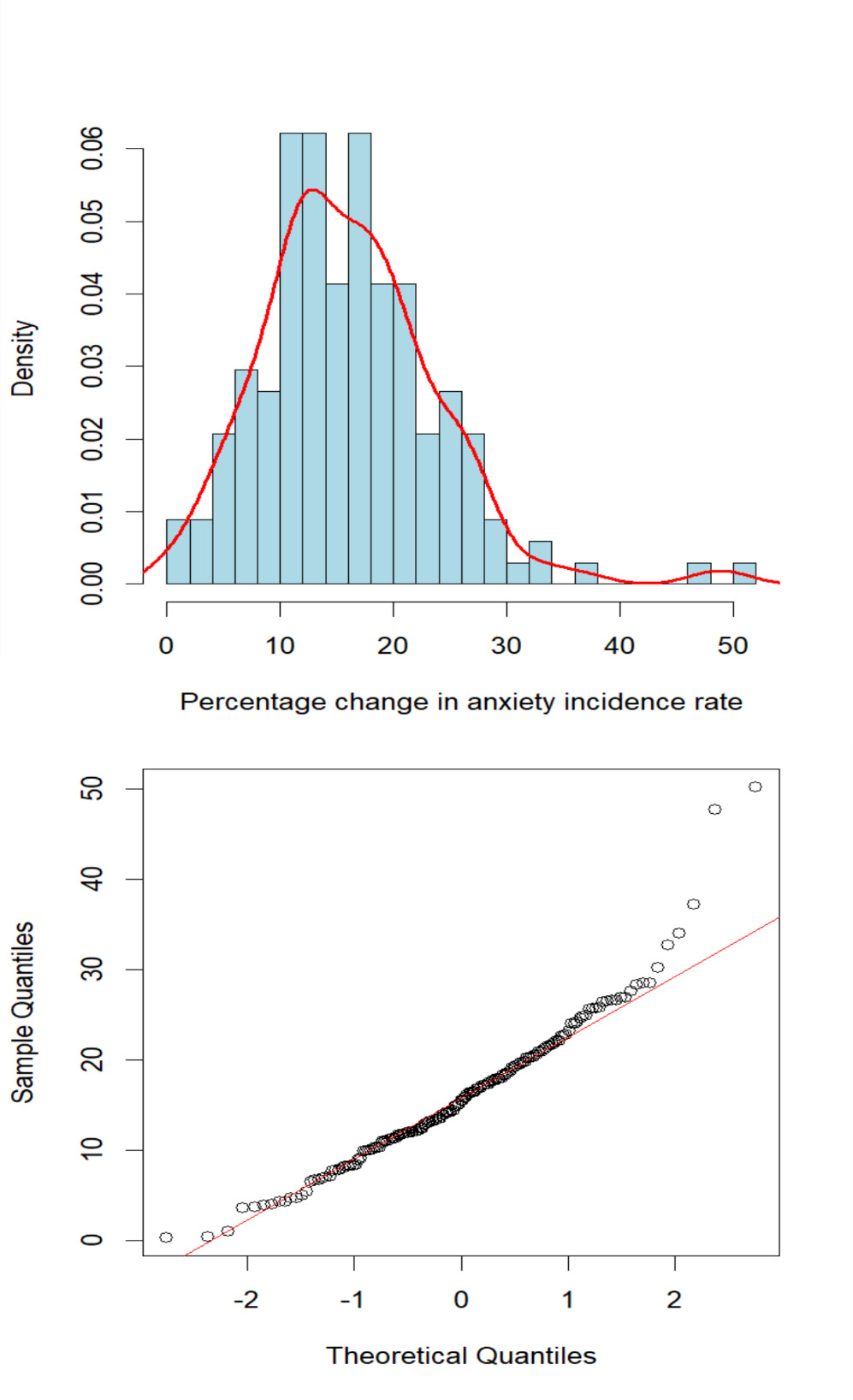
**

**Supplementary figure A.1** Histogram and Q-Q plot for percentage change in incidence rates of anxiety disorders (2019-2020)

**
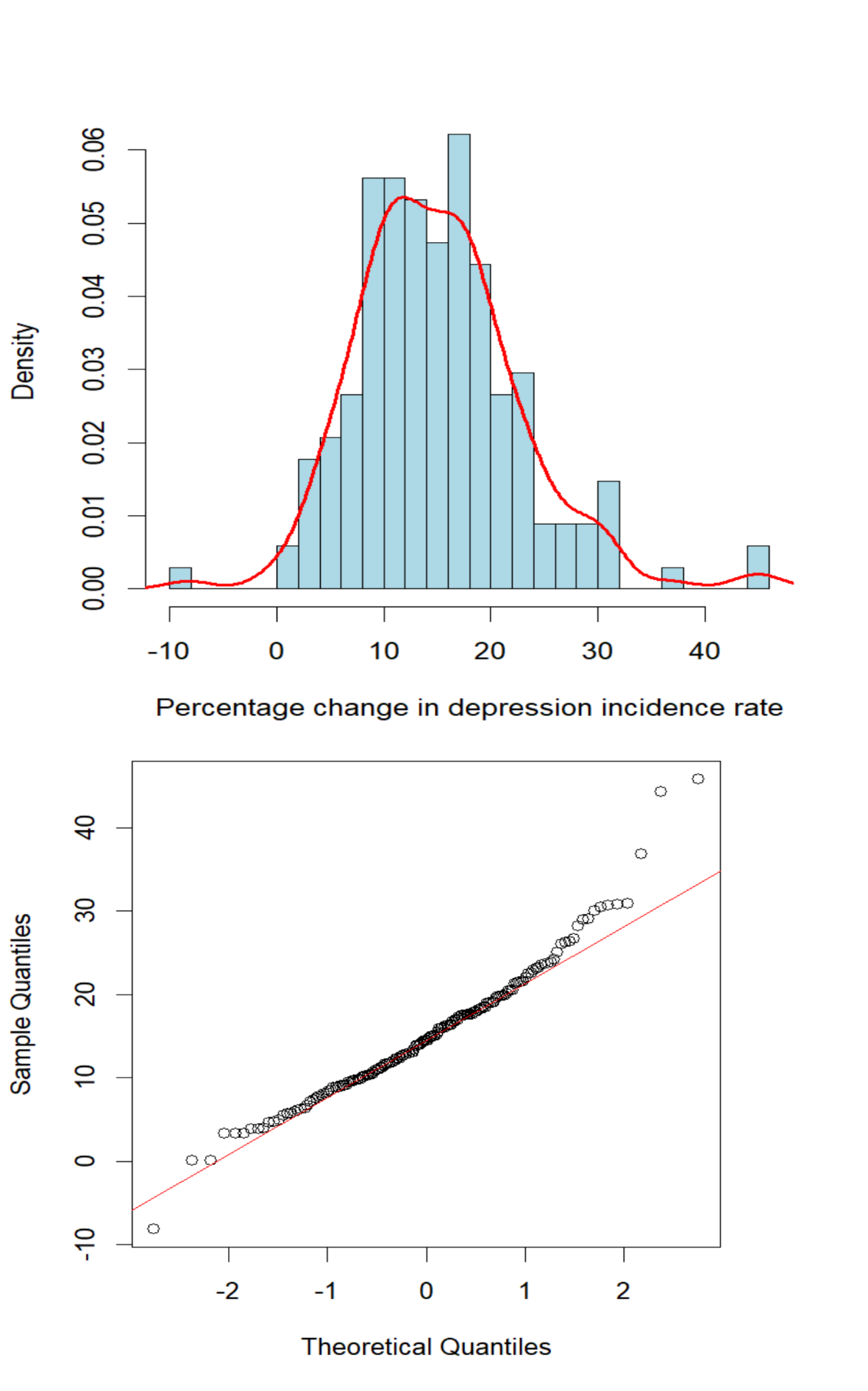
**

**Supplementary figure A.2** Histogram and Q-Q plot for percentage change in incidence rates of depressive disorders (2019-2020)

**
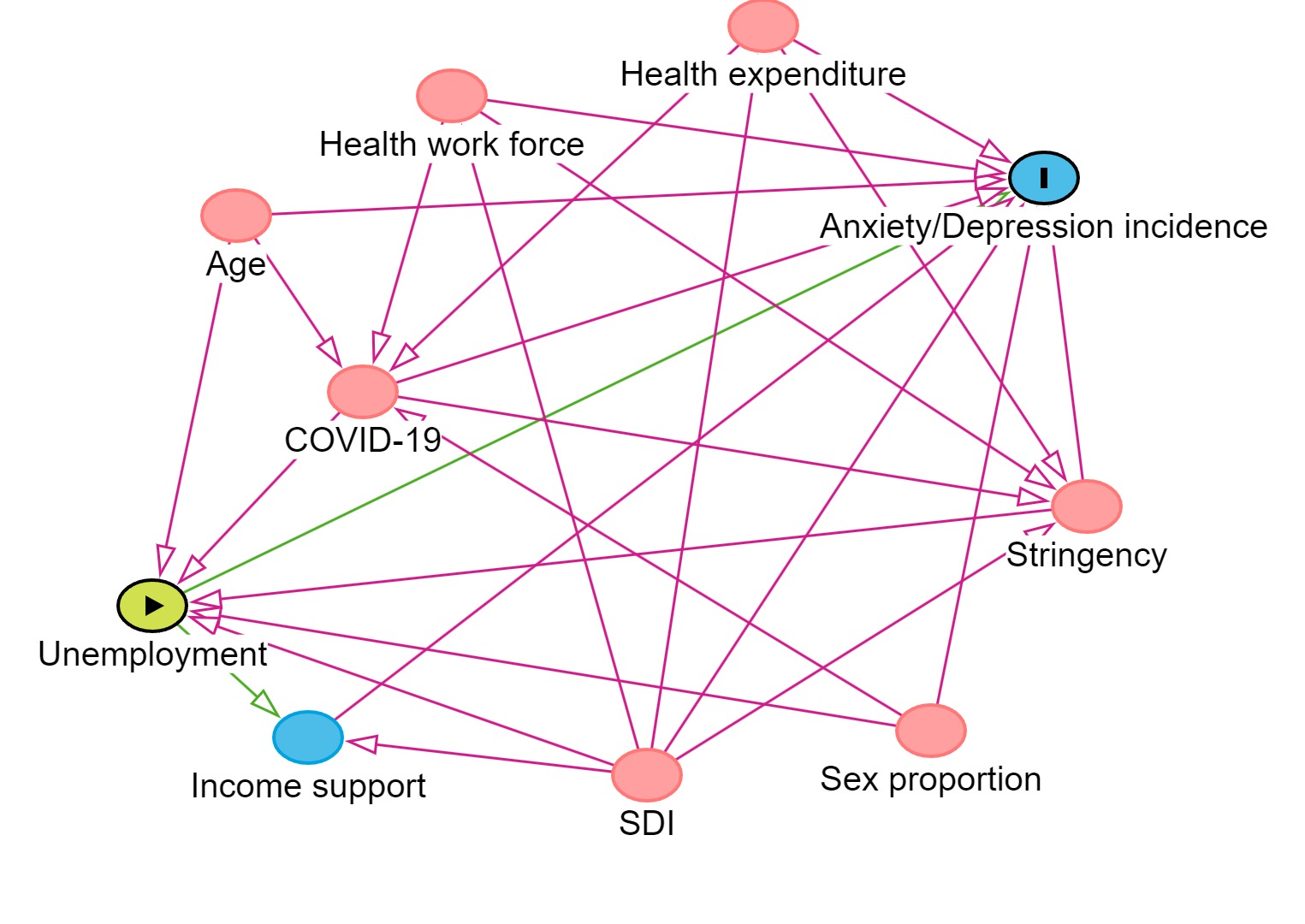
Supplementary figure A.3** A directed acyclic graph representing a possible causal model for the association between unemployment and mental disorders

**
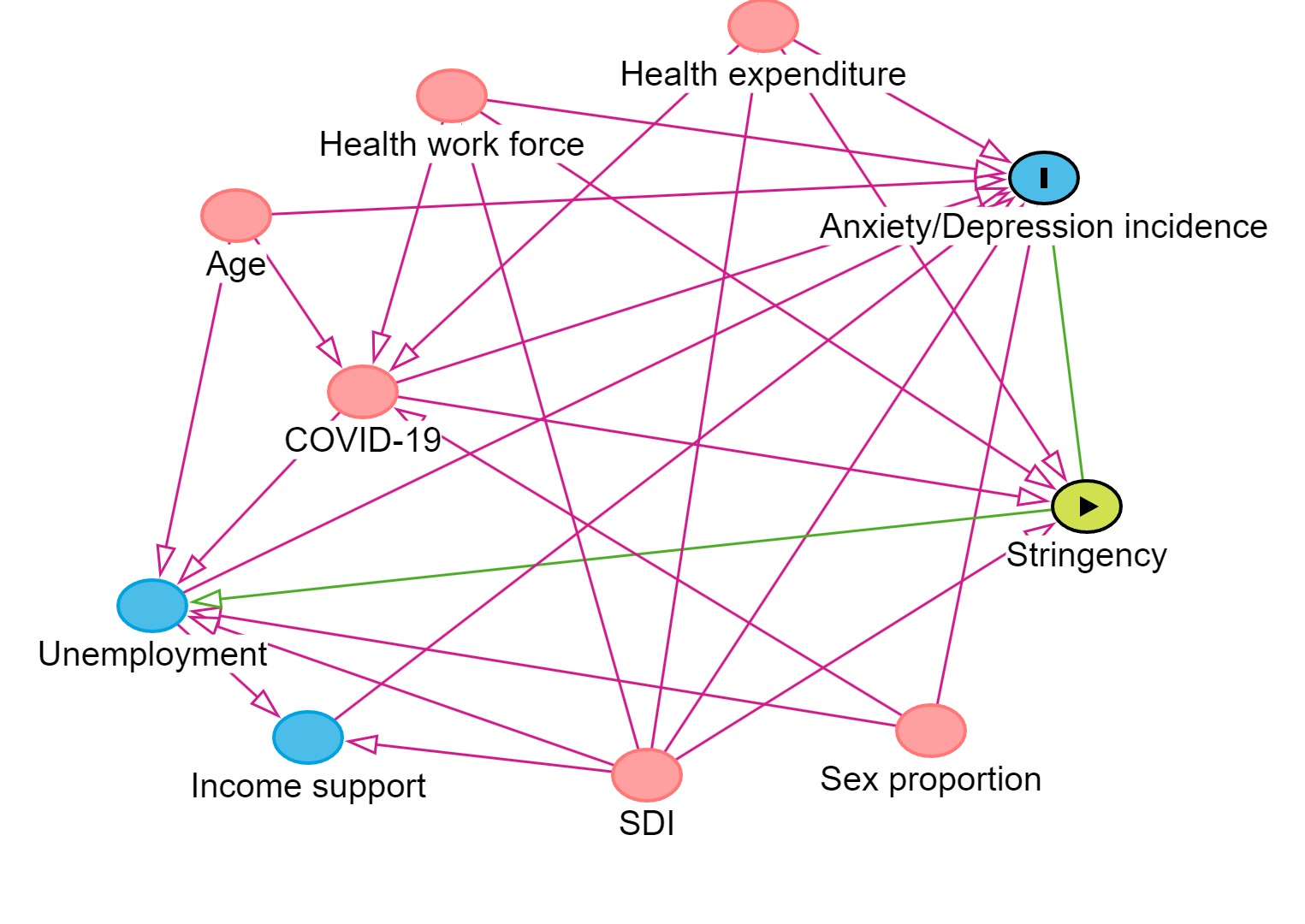
**

**Supplementary figure A.4** A directed acyclic graph representing a possible causal model for the association between government stringency and mental disorders

**
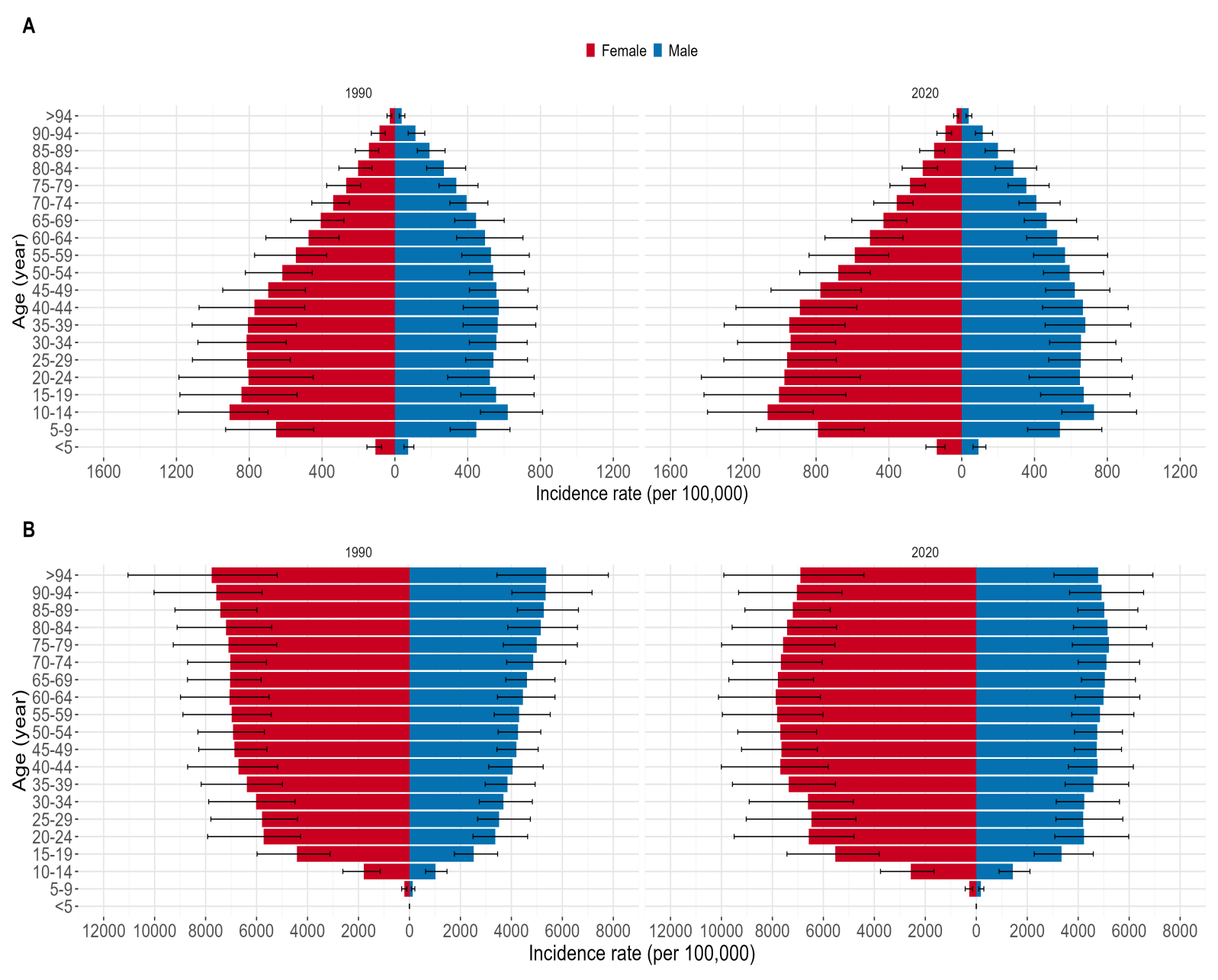
**

**Supplementary figure A.5** Incidence rate by age and sex. (A) Anxiety disorders in 1990 and in 2020; (B) Depressive disorders in 1990 and in 2020.

**
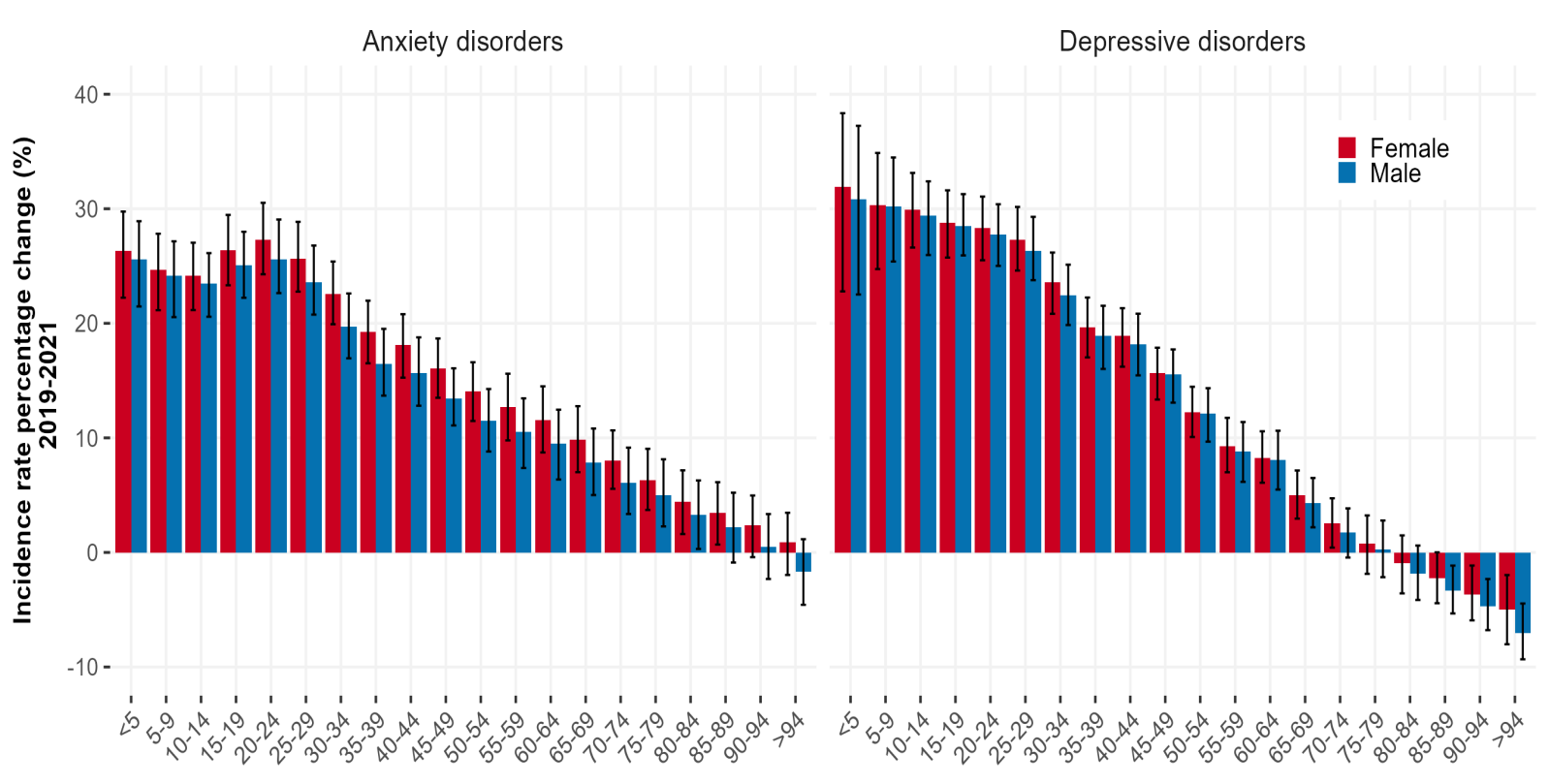
**

**Supplementary figure A.6** Relative changes in incidence rate of anxiety and depressive disorders from 2019 to 2021.

## **Supplementary figure A.7** Geographic distribution of age-standardized incidence rate and age-standardized YLD rate of anxiety disorders in 1990, 2000, 2010, 2019, and 2021

YLD = years lived with disability.
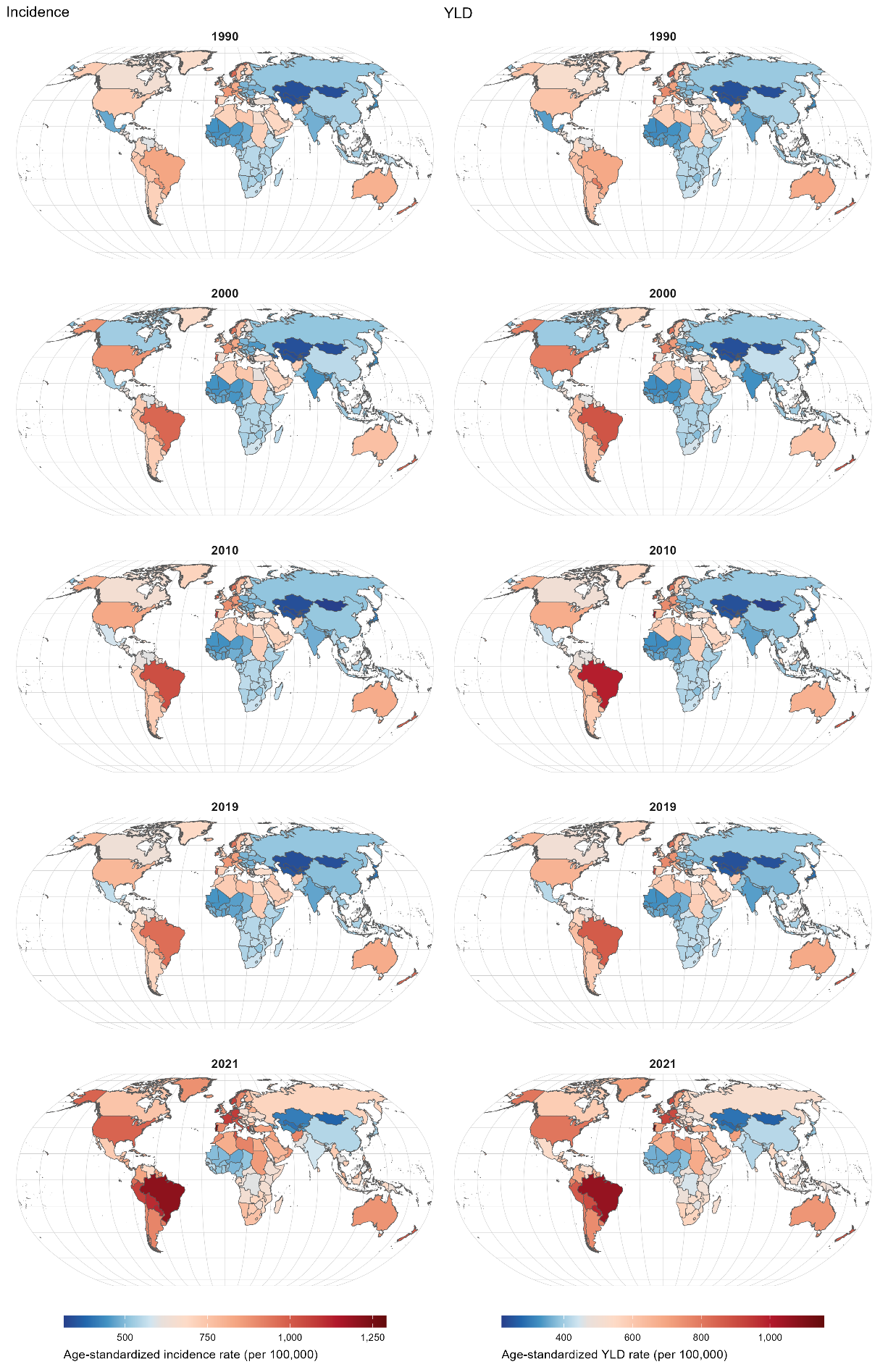


**
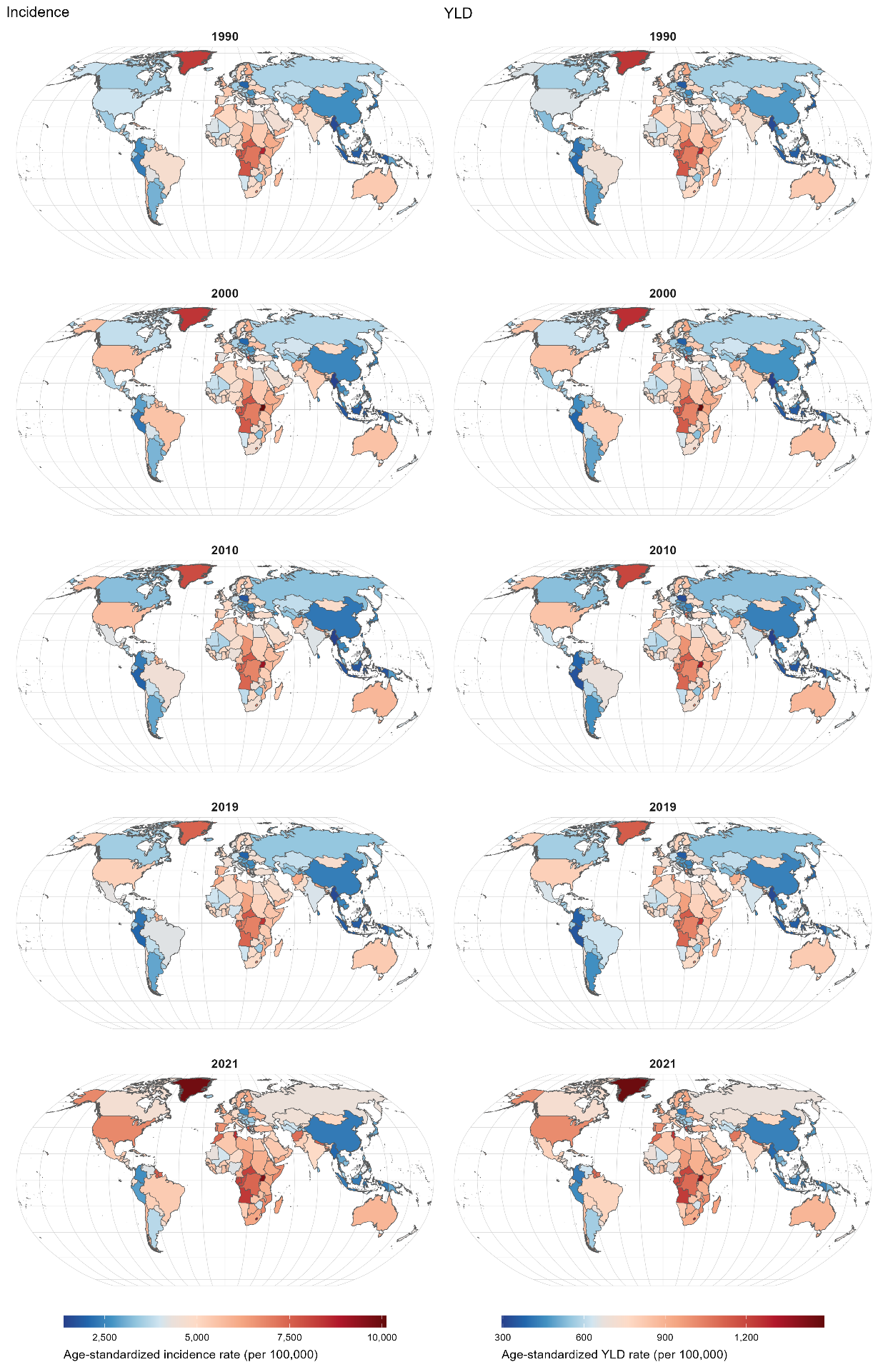
**

## **Supplementary figure A.8** Geographic distribution of age-standardized incidence rate and age-standardized YLD rate of depressive disorders in 1990, 2000, 2010, 2019, and 2021

YLD = years lived with disability.

**Supplementary figure A.9** Spearman’s correlation matrix among percentage change in incidence rates of anxiety disorders and depressive disorders, socioeconomic indicators, and government response policies during the COVID-19 pandemic.

CPI=consumer price index; GDP=gross domestic products

Note: All changes indicated changes from 2019 to 2020. Socio-demographic index (SDI) was the mean values of SDI of each country and territory in 2020. Economic support index and government stringency index were the median values of each county and territory throughout 2020. COVID-19 testing policies, COVID-19 contact tracing policies, face covering policies, public events cancellation, public gathering cancellation, public transport restrictions, school closures, and workplace closures were the mode values throughout 2020.

**
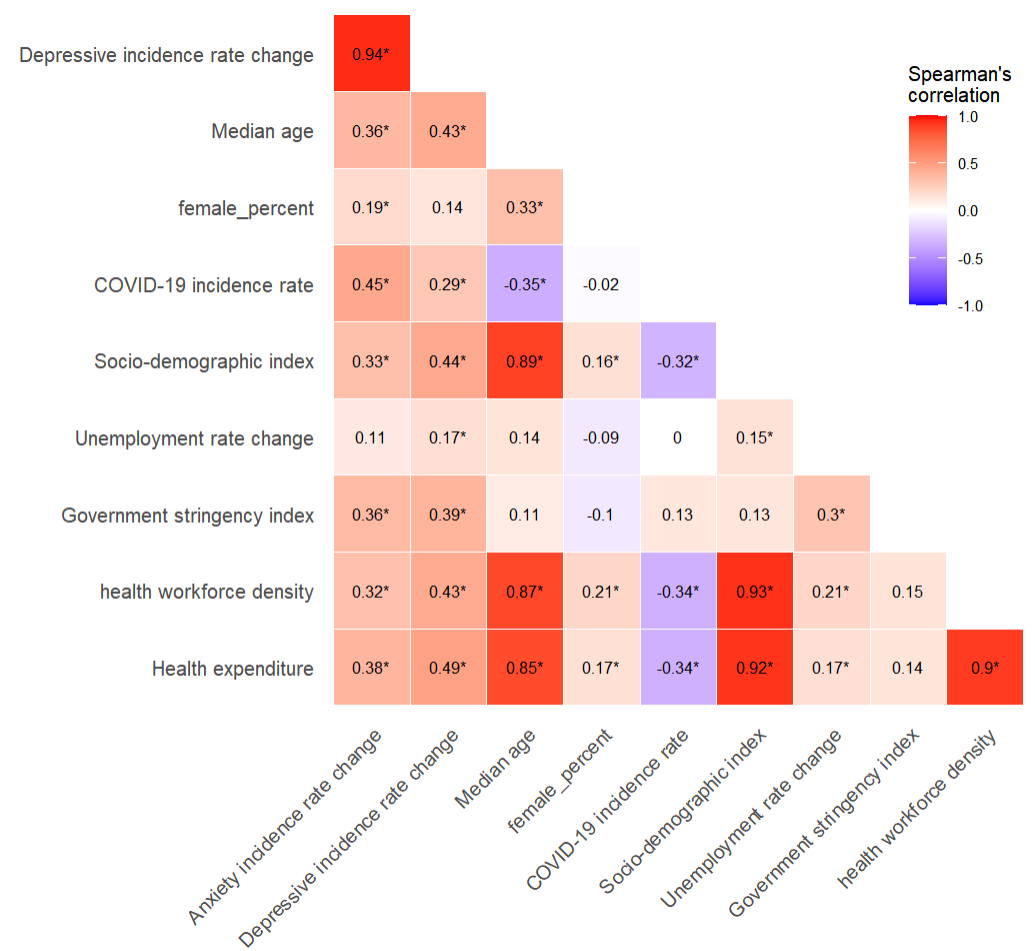
**

**Supplementary figure A.9** Spearman’s correlation matrix among percentage change in incidence rates of anxiety disorders and depressive disorders and socioeconomic indicators during the COVID-19 pandemic.

Note: All changes indicated changes from 2019 to 2020.
